# The Effect of Protandim^®^ Supplementation on Athletic Performance and Oxidative Blood Markers in Runners

**DOI:** 10.1371/journal.pone.0160559

**Published:** 2016-08-11

**Authors:** Seteena L. Ueberschlag, James R. Seay, Alexandra H. Roberts, Pamela C. DeSpirito, Jeremy M. Stith, Rodney J. Folz, Kathleen A. Carter, Edward P. Weiss, Gerald S. Zavorsky

**Affiliations:** 1 Department of Health and Sport Sciences, University of Louisville, Louisville, KY, United States of America; 2 Department of Medicine, University of Louisville, Louisville, KY, United States of America; 3 Department of Clinical Sciences, University of Kentucky, Lexington, KY, United States of America; 4 Department of Medicine, Case Western Reserve University, Cleveland, OH, United States of America; 5 Department of Nutrition and Dietetics, Saint Louis University, Saint Louis, MO, United States of America; 6 Department of Respiratory Therapy, Georgia State University, Atlanta, GA, United States of America; Universidade do Extremo Sul Catarinense, BRAZIL

## Abstract

The purpose of this study determined if oral supplementation of Protandim^®^ (a nutraceutical) for 90 days improved 5-km running performance and reduced serum thiobarbituric acid-reacting substances (TBARS) at rest, an indicator of oxidative stress. Secondary objectives were to measure whole blood superoxide dismutase (SOD), glutathione (GSH), and glutathione peroxidase (GPX), at rest and 10 minutes after completion of the race before and after supplementation as well as quality of life. In a double-blind, randomized, placebo controlled trial, 38 runners [mean (SD) = 34 (7) yrs; BMI = 22 (2) kg/m^2^] received either 90 days of Protandim^®^ [1 pill a day, n = 19)] or placebo (n = 19). Randomization was done in blocks of two controlling for sex and 5-km baseline performance. A 5-km race was performed at baseline and after 90 days of supplementation, with blood samples taken before and 10-min after each race. Fasting blood samples were acquired at baseline, after 30, 60, and 90 days of supplementation. TBARS, SOD, GPX, and GSH were assayed in an out-of-state accredited lab. Running performance was not altered by Protandim^®^ or placebo [20.3 (2.1) minutes, with an -8 (33) seconds change in 5-km time regardless of group]. There was no change in TBARS, SOD, or GPX (at rest) after three months of Protandim^®^ supplementation compared to placebo. However, in a subgroup ≥ 35 years of age, there was a 2-fold higher increase in SOD in those taking Protandim^®^ for three months compared to those on placebo (*p* = 0.038). The mean post-race change in TBARS (compared to pre-race) increased by about 20% in half of the subjects, but was not altered between groups, even after three months of supplementation. Quality of life was also not different between the two conditions. In conclusion, Protandim^®^ did not (1) alter 5-km running time, (2) lower TBARS at rest (3) raise antioxidant enzyme concentrations compared to placebo (with exception of SOD in those ≥ 35 years old) or, (4) affect quality of life compared to placebo.

***Trial Registration*:** ClinicalTrials.gov NCT02172625

## Introduction

Cells continuously produce free radicals (a molecule with one of more unpaired electrons) and non-radical derivatives of oxygen (i.e. hydrogen peroxide) as part of metabolism. Free radicals and non-radical derivatives of oxygen are collectively termed reactive oxygen species (ROS) [1]. Reactive oxygen species are associated with aging cells [2], and can also increase acutely after exercise, as demonstrated by lipid peroxidation in serum samples obtained from blood [3–7]. Thus, oxidative stress occurs when the production of reactive oxygen species (ROS) outweighs the body's ability to detoxify them [8].

Reactive oxygen species is neutralized by a sophisticated antioxidant defense system consisting of antioxidant enzymes such as superoxide dismutase (SOD), glutathione peroxidase (GPX), and catalase [9]. Superoxide dismutase is the body's first line of enzymatic defense against intracellular free radical production through reduction of one-electron dismutation of oxygen (O_2_^-^) to hydrogen peroxide (H_2_O_2_) [10]. Non-enzymatic antioxidants like Vitamins A (beta-carotene), E, and C also protect every cell in our body from free radical damage [10].

While the generation of ROS is a by-product of cellular respiration, oxidative stress, as demonstrated by serum levels of lipid peroxides, is shown after single bouts of exercise [3–7], but regular endurance training may reduce lipid peroxidation [3]. In addition, physical overtraining can also increase oxidative stress. Three weeks of six days per week high intensity resistance training increased oxidative stress in blood as indicated by serum thiobarbituric acid-reacting substances (TBARS), which is an indicator of lipid peroxidation, by 56% while reducing whole blood total glutathione content (GSH) by 31% (an endogenous antioxidant), and total antioxidant capacity (TAC) by 20% [11]. A lower TAC suggests increased oxidative stress. As a result of increasing oxidative stress, athletes experience greater fatigue, muscle damage, and increased recovery time [12]. However, individuals who perform endurance training on a regular basis increase SOD and GPX by 25–35% within the muscle [13], and up to 45% in blood [11].

Protandim^®^ marketed by LifeVantage Corporation, is a nutritional supplement comprised of five plant extracts (milk thistle, bacopa, ashwagandha root, turmeric, green tea) that supposedly activates the Nuclear factor (erythroid-derived 2)-like 2, (called Nrf2) transcription factor pathway that is integral to several antioxidant enzymes, including γ-glutamyl cysteine synthase (an enzyme that catalyzes the committed step in glutathione synthesis) [14]. Nuclear factor (erythroid-derived 2)-like 2 is a basic leucine zipper protein transcription factor that regulates the expression of antioxidant proteins that protect against oxidative damage triggered by injury and inflammation.

In the past 10 years, there have been several studies published that used Protandim^®^ [15–21] but only three were assessed in human subjects [15, 16, 21]. In those human studies, the results were mixed, with some showing that oxidative damage was reduced with long term supplementation [15, 21] and another showing no reduction in oxidative damage [16]. Even so, a study showing that oxidative damage was reduced after a month of supplementation did not include a placebo group [15].

To date, there are no studies evaluating the effects of Protandim^®^ supplementation on endurance performance or the attenuation of oxidative damage from endurance exercise. By supplementing regional-class runners with Protandim^®^ (675 mg/day), this study seeks to answer whether ~90 days of Protandim^®^ supplementation improves 5-km running performance and reduces serum TBARS at rest (an indicator of oxidative stress). A secondary objective was to look at blood antioxidant enzymes (GPX, SOD) at rest and 10 minutes after the completion of the run before and after the supplementation period. Thus, the main outcome measures were 5-km running time and TBARS. Secondary outcome measures were SOD, GPX, quality of life, and other blood variables such as whole blood glutathione, total antioxidant capacity, sulfate, cysteine and cystine.

## Methods

### Participants

Forty runners, 20 to 46 years of age were recruited from running clubs across the Louisville, Kentucky, community between November 2014 and February 2015, with data collection completed in May 2015. The runners had to be considered “local class” or faster for 5-km time, based on United States Track-and-Field age and sex graded performance categories. All participants were asked to abstain from taking any nutritional supplements including: vitamins, minerals, and any over the counter products. Participants were allowed to take ferrous sulfate, elemental iron, vitamin D, and calcium. Subjects were excluded prior to the start of the study if they had any known allergy to milk thistle, bacopa monnieri, ashwagandha, tumeric (ginger), tea, caffeine, tannins, or members of the theaceae family. All subjects provided written informed consent. The study was approved by the University of Louisville Institutional Review Board (IRB number: 14.0614, S1 Protocol).

### Procedures

This study was a double-blind, randomized, placebo controlled trial where participants were placed into one of two groups randomly by blocks of two, controlling for sex and running time (clinicaltrials.gov identifier: NCT02172625, and S1 Checklist). After the completion of session two, principal investigator and co-author G.S.Z. generated the random allocation sequence through the use of a coin toss, and assigned the participants to two groups while graduate students and co-authors S.L.E. and J.R.S. observed and recorded the allocation. The experimental group took the supplement Protandim^®^ for the entire duration of the study (1 capsule of 675 mg/day containing 225 mg of milk thistle, 150 mg of bacopa, 150 mg dose of ashwagandha root, 75 mg of turmeric, and 75 mg green tea, US Protandim^®^ Lot# X14-0901). The control group took a placebo (corn starch and food coloring) presented in identical green capsules. The two different types of capsules (Protandim^®^, Corn Starch) were coded by LifeVantage Corporation such that all participants, all co-authors (including the principal investigator G.S.Z.), and the clinical chemistry laboratory did not know which subject was receiving Protandim^®^ or corn starch. Each subject underwent six testing sessions, with five different days of blood draws.

#### Session 1 (initial screening day)

Subjects were instructed to complete a physical activity readiness questionnaire to clear them for physical activity. They were also asked to refrain from taking any multivitamins or nutritional supplements for the duration of the study due to the previous evidence that Vitamin E and C supplementation affects plasma TBARS [15]. A training diary was given to each participant to fill out for the entire duration of the study and were asked to use the diary to record their intensity, training duration, and mileage per week of all aerobic-type activities. A 24-hr dietary recall form was also provided to the subjects. Lastly, each participant was given five quality of life (QOL) questionnaires (WHOQOL-BREF) to fill out at home for the duration of the study [22]. Quality of Life is scored in four domains: physical health, psychological, social relations, and environment [22].

#### Session 2 (baseline, 15 days after Session 1)

Subjects were fasted in the morning when they arrived at University of Louisville for their pre-exercise blood sampling. Each subject brought their 24-hr dietary recall to the session as well as their QOL form [22]. Approximately 15 mL of blood was withdrawn 30 minutes prior to exercise for analysis of several blood parameters. Following blood samples being taken, participants partook in the first of two baseline 5-km time trials at the University of Louisville outdoor track. Time trials have greater logical validity compared to time-to-exhaustion tests [23]. Approximately 10-minutes post-exercise, another 15 mL of blood was taken from each subject. Only after their post-exercise blood draw were subjects allowed to eat. From the results of the first 5-km time trial, the participants were divided into one of two groups based on their results. The participants were randomly assigned in blocks of two so that number of males and females per group would be similar and the average 5-km performance time per group would be similar.

#### Session 3 (7 days after Session 2)

Subjects were fasted in the morning when they arrived for their pre-exercise blood sampling. Each subject brought their 24-hr dietary recall to the session as well as their QOL form [22]. Blood was withdrawn 30 minutes prior to exercise, and then each subject was required to partake in another baseline 5-km time trial on the outdoor track. The reason for a second baseline 5-km time trial was to assess the week-to-week coefficient of variation in this group of runners. Approximately 10 minutes post-exercise, blood was withdrawn again. Only after their post-exercise blood draw were subjects allowed to eat. Then, depending on the group, subjects were given either a ~90 day supply of Protandim^®^ pills or placebo pills. Since the study was double-blinded, neither the researchers nor the subjects knew which pills they were ingesting. Subjects were instructed to ingest one pill per day, ideally with breakfast (675 mg per day, for ~90 days). The subjects were also given a signs/symptoms form, where they were asked to report any signs/symptoms they had during the supplementation period, such as diarrhea, stomach aches, nausea, etc.

#### Sessions 4 and 5 (~30 days and 60 days post-supplementation)

Participants arrived fasted in the morning for their pre-exercise blood sampling. All forms were collected by a member of the research team and there was a blood draw. There was no 5-km running race performed at this session.

#### Session 6 (~90 days post-supplementation)

Participants arrived fasted in the morning for their pre-exercise blood sampling. They each brought their 24-hr dietary recall with them, their QOL form [22], their running logs, and their signs/symptoms form. Participants also brought with them any unused pills for proper documentation. Following participants’ blood being drawn, each subject was required to partake in their final 5-km time trial at the University of Louisville outdoor track. At approximately 10-minutes post-exercise, blood was withdrawn from each subject.

### Blood sampling

Blood was collected in vacuum-sealed tubes designed to contain and preserve specimens in a manner appropriate for their respective analysis. Once drawn, specimens were separated and the ethylenediaminetetraacetic acid (EDTA) tubes were placed at 4°C, whereas the larger gel tubes were allowed to clot for fifteen minutes and then centrifuged at 3000 revolutions per minute (Champion F-33 Series, Ample Scientific, Norcross, GA) for fifteen minutes. After centrifugation, the serum samples were frozen at -20°C for at least 4 hours and then shipped overnight to Geneva Diagnostics for analysis using proprietary methodology (Oxidative Stress Analysis 2.0, Blood). Genova Diagnostics is a global, fully accredited clinical laboratory, located in Asheville, North Carolina [Licensed by Clinical Laboratory Improvement Amendments (CLIA) Certification number #34D0655571]. The blood was analyzed for what follows.

1. **Serum TBARS.** This is a direct biomarker of total serum lipid peroxidation, which are the products of the chemical damage done by oxygen free radicals to the polyunsaturated fatty acids of cell membranes. Serum TBARS have been shown to be correlated with oxidative damage to certain tissues, namely heart and liver tissue at rest (Pearson Product Moment Correlation, or r = 0.71 to 1.0) and exercise (r = 0.68 to 0.99) [24]. The Lipid Peroxide assay is designed to measure the lipid peroxidation products in serum. After acid hydrolysis, the lipid peroxidation products are reacted with thiobarbituric acid resulting in a spectrophotometrically active product. Malondialdehyde is used as the standard for determination of levels of lipid peroxidation products.
2. **Total antioxidant capacity (TAC).** The TAC measures the overall collective power of the blood to neutralize free radicals. Specifically, the TAC assay measures the antioxidant capacity of a serum sample via the ability of the antioxidants within the sample to neutralize a spectrophotometrically active compound that is optically active when oxidized. The decrease in color intensity of the compound when compared to the standard, Trolox, under the same reaction conditions is equivalent to the serum antioxidant capacity of the serum sample.
3. **Whole blood total glutathione content.** This includes both reduced and oxidized states together, is the most potent endogenous antioxidant, is correlated well with muscle and heart tissue at rest (r = 0.93 to 1.0) [24] and at exercise (r = 0.66 to 1.0) [24], and the assay is designed to move all to the reduced form of measurement so GSH becomes total reduced GSH content in whole blood (ie., GSH+ GSSG + GSSProt). The total whole blood glutathione assay is designed to measure the level of glutathione in whole blood. The samples is first completely lysed and proteins are precipitated. The supernatant is then reduced and combined with a spectrophotometrically reactive compound which generates a detectable absorption peak. When compared to known concentrations of glutathione under the same reaction conditions a determination of glutathione levels in blood is determined.
4. **Superoxide Dismutase (SOD).** This is another protective antioxidant enzyme measured from whole blood. The SOD enzymatic assay from Genova Diagnostics is designed to measure the activity of the SOD enzyme in the cytosol. The SOD assay is designed to measure the activity of superoxide dismutase enzyme (SOD) from whole blood. The SOD activity is determined spectrophotometrically based on the ability of the superoxide dismutase compound to reduce reactive oxygen species in an enzymatic reaction necessary for the production of an optically active compound. The result is expressed as units of SOD relative to the gram amount of hemoglobin in the sample.
5. **Glutathione peroxidase (GPX).** This is a measure of glutathione peroxidase activity in red blood cell lysates sampled from whole blood. The level of GPX in the sample is determined spectrophotometrically based on the ability of the compound to catalyze a reduction reaction in the presence of glutathione. The change in the absorption level of the substrate is then utilized to determine the level of GPX present in the sample. The result is expressed as units of GPX relative to the gram amount of hemoglobin in the sample.
6. **Serum sulfate.** This is produced from cysteine via sulfoxidation, and a critical factor in detoxification reactions. The assay designed for determination of sulfate levels in serum is a turbidimetric assay utilizing the chemical property of sulfate ions to cause the formation of precipitates that can be measured by absorbance of light. The use of a sulfate standard curve under the same reaction conditions facilitates the ability to determine the level of sulfate in the serum sample.
7. **Serum cysteine to sulfate ratio.** This is a reflection of the efficiency of the conversion of cysteine to sulfate. The assay designed for the measurement of serum cysteine is an adaptation of the Gaitonde procedure developed for the detection of amino acids which utilizes the colorimetric reaction of amino acids with ninhydrin.
8. **The serum cysteine to cystine ratio.** Cystine is oxidized disulfide form of cysteine. The serum cysteine to cystine ratio is a measure of the redox balance in serum.
9. **Blood glucose.**

### Sample size calculation

The main independent variable in this study was the two different supplementation groups. The main dependent variables measured were 5-km finishing time, TBARS, GSH, SOD, GPX, TAC, and the four domains of the QOL form. Based on a 5-km time improvement of 2.5% or about 30 seconds (SD = 1 minute) with Protandim^®^, and no improvement in the placebo group, about 34 runners in total were needed (Effect size for ANOVA = *f* = 0.25, which is moderate, statistical power = 80%, two-sided alpha error probability = 5%, two measurements per group, two groups, correlation amongst repeated measures = 0.50, F-test Family, ANOVA repeated measures, within-between interaction, G*Power 3.1.2, Universität Kiel, Germany). Accounting for a ~20% attrition rate (8 subjects), a total of 40 subjects were recruited (20 per group).

### Statistical analyses

A 2 x 4 mixed design analysis of variance (ANOVA) was chosen to compare long term, chronic changes in blood parameters (rested, fasted state) over the duration of the study (2 groups; 4 time-points: average baseline, 30, 60, 90 days post-supplementation). It is a mixed ANOVA as there is a mixture of between groups and repeated measures variables. Another 2 x 4 mixed design ANOVA compared the acute changes in blood parameters between pre and immediately post exercise at before and after the supplementation period (2 groups; 4 time-points: average of both baseline pre-exercise values, average of both baseline post-exercise values, then 90 days post supplementation pre-exercise, and 90 days post supplementation post-exercise). When sphericity was not achieved, a Greenhouse-Geisser adjustment was used. This design also provided the experimenter the opportunity to control for individual differences among participants. To adjust for multiple comparisons *post-hoc*, the Benjamini-Hochberg procedure was used since it provides better statistical power than the Bonferroni correction [25].

To compare groups, baseline subject characteristics (including anthropometric data, environmental conditions, and resting, fasted, blood variables) were performed using independent *t*-tests. If any of the variables were not normally distributed (as verified by a Shapiro-Wilk test), then a Mann-Whitney *t*-test was used to compared groups.

We determined the inter-session variability over time in order to distinguish between the inherent variability of the test, from small, real physiological change caused by an interventional study. To compare the variability in baseline 5-km time-trials from week-to-week, the coefficient of variation (C.V.) was calculated for each subject and averaged [(SD ÷ mean)·100]. To calculate the reproducibility in 5-km times, the following was done: Reproducibility was calculated by obtaining the square root of the mean square error obtained from a repeated measures analysis of variance obtained from the two 5-km baseline time trials that were performed in a one week period. Both groups were placed together in this analyses because neither group as of that point was under the influence of the supplement. The square root of the mean square error obtained from the repeated measures ANOVA was reported as the common week-to-week within subject standard deviation (SD_w_) [26]. Reproducibility was defined as 2.77·SD_w_ [26]. That is, the difference between the 5-km times on different weeks for the same subject is expected to be less than 2.77 times the within-subject standard deviation for 95% of pairs of observations [26]. Since the calculation of reproducibility may be considered too stringent, the smallest measureable change was reported as half of the reproducibility [27]. Any 5-km time that was above or below the smallest measureable change was considered a meaningful change. As well, the C.V. and reproducibility was calculated for each domain of the QOL form. Furthermore, the baseline C.V. was calculated for all blood parameters.

The baseline 5-km time was reported as the as the fastest of the two baseline 5-km time trials. A Fisher’s exact test determined whether there was a difference between the two groups in the number of subjects that improved by more than the smallest meaningful change in 5-km time post-supplementation. A Fisher’s exact test was also used to compare groups for the signs and symptoms reported during the supplementation period.

In order to control for differences in the exercise training regimes between the two groups, an aerobic training index was calculated at baseline, and then again post-supplementation. The aerobic training index was calculated as the total number of minutes of aerobic physical activity over the previous 14 days multiplied by the average rating of perceived exertion score over the previous 14 days (6 = no exertion, 20 = maximal exertion. A Kruskal-Wallis one-way analysis of variance was used to compare the training index between groups pre and post supplementation. A Kruskal-Wallis one-way analysis of variance was also used to compare each domain of the WHOQOL-BREF between groups during the supplementation period. The WHOQOL-BREF scores within each domain was adjusted for multiple comparisons using the Benjamini-Hochberg procedure.

Statistical significance was set at 0.05. Statistical analyses was performed using IBM SPSS for Windows version 21.0, released in 2012 (IBM Corporation, Armonk, NY).

## Results

**Fig 1:**
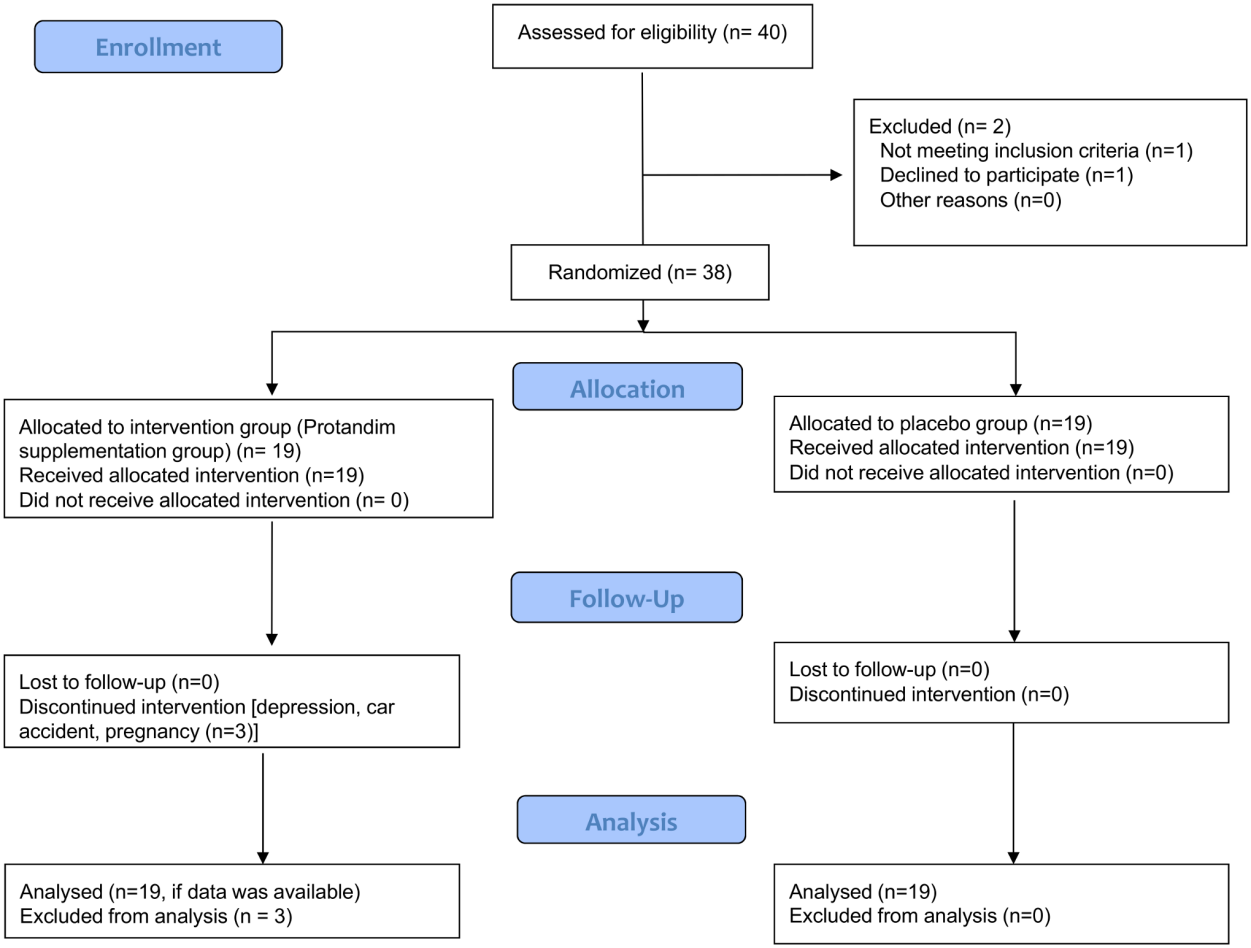
Flow diagram describing enrollment, allocation, follow-up, and analysis of this clinical trial.

One subject did not make the time-trial standard so she was eliminated from the study, and the other subject did not continue on with the study after the informal information session. Thus, 38 subjects (20 men, 18 women) were retained (Fig 1). The anthropometric characteristics, baseline 5-km times, and fasting blood glucose concentration were not different between groups, as shown in Table 1.

**Table 1:** Baseline anthropometric characteristics, baseline 5-km time-trial results, and baseline fasting blood glucose results.

Variables	Protandim^®^	Placebo	*p* -value	Combined Mean
	(n = 19)	(n = 19)		(n = 38)
Age (yrs)	34 (6)	35 (8)	0.56	34 (7)
	[23 to 44]	[20 to 46]		[20 to 46]
Weight (kg)	68.1 (11.4)	64.6 (11.1)	0.34	66.4 (11.2)
	[42.6 to 88.6]	[47.5 to 88.5]		[42.6 to 88.6]
Height (cm)	174 (10)	171 (11)	0.30	172 (11)
	[155 to 191]	[155 to 188]		[155 to 191]
BMI (kg/m^2^)	22.3 (2.1)	22.1 (2.7)	0.82	22.2 (2.4)
	[17.7 to 26.3]	[18.6 to 27.6]		[17.7 to 27.6]
5-km time (sec)	1225 (136)	1210 (121)	0.72	1217 (128)
	[1047 to 1450]	[1029 to 1469]		[1029 to 1469]
5-km time (min)	20.4 (2.3)	20.2 (2.0)	0.72	20.3 (2.1)
	[17.5 to 24.2]	[17.2 to 24.5]		[17.2 to 24.5]
% of world record for age & gender	68 (4%)	69 (6%)	0.34	69 (5%)
	[61% to 76%]	[60% to 81%]		[60% to 81%]
Fast blood glucose (mg/dL)	90 (5)	89 (9)	0.73	90 (7)
	[81 to 97]	[73 to 104]		[73 to 104]

Mean (SD), [range], 22 subjects (58% of the sample) was classified as Local Class, 15 subjects (40% of the sample) was classified as Regional Class, one subject (3% of the sample) was classified as National Class. Baseline 5-km time trial performance was taken as the best result between two baseline 5-km time trials held one week apart. Baseline fasting blood glucose values was the average fasting blood glucose values for both baseline sessions held one week apart. All variables were normally distributed (Shapiro-Wilk test *p* > 0.05 for all).

In the end, one female subject withdrew from the study two weeks after the beginning of supplementation due to complaints that the supplement caused her to be depressed. It was later determined that she was in the Protandim^®^ group. In addition, another female subject dropped out just before the final 5-km time trial because she was pregnant. A male subject also did not complete the final 5-km time trial because he was in a car accident a week earlier. However, he did have his fasted pre-exercise blood drawn at ~88 days supplementation.

Venous blood samples were obtained at baseline (there were two baseline sessions one week apart from each other), and at 30 (SD 2), 57 (2), and 88 (4) days post-supplementation. The subjects returned their pill bottles and the combined average number of pills not taken by both groups was 3 (4), with a range of 0 to 19 pills missed.

### 5-km time trial performance

These 38 subjects were randomized into the experimental and control groups by random blocks of two according to gender and 5-km time trial performance. In the end, equal number of males and females were in the Protandim^®^ group and the Placebo group, and both groups had similar 5-km times of ~20.3 (2.1) minutes (Table 1). The mean rating of perceived exertion (RPE) for the baseline 5-km time trials was 17.5 (1.7) out of 20 (6 = no exertion, 20 = maximal exertion). This mean value is qualitatively labeled between “very hard” and “very very hard” for the effort of the 5-km runs. The data presented in Table 1 is the best performance of both baseline time-trials. The mean coefficient of variation between both baseline 5-km time trials was 1.1% and the correlation between both these 5-km time trials was 0.99, (*p* < 0.01). There was no difference in mean 5-km time trial performance between both baseline sessions. After removal of outliers, the reproducibility was 23 seconds, and the smallest meaningful change was 12 seconds (half of the reproducibility).

Changes in training status over the supplementation period was similar between groups as assessed by the aerobic training index (*t* = -1.63, *p* = 0.11). Thus, if there were any changes in running performance observed between groups over the three month period, it would be more likely due to a supplementation effect compared to a training effect. Overall, running time was not altered by Protandim^®^ or placebo supplementation [20.3 (2.1) minutes, with an -8 (33) seconds change in 5-km time regardless of group [*p* = 0.19 between baseline and 88 days post supplementation, and *p* = 0.91 between Groups, and *p* = 0.83 for the Group x Time interaction effect). There were eight out of 16 subjects that improved by ≥ 12 seconds in the Protandim^®^ group, while nine out of 19 subjects improved by ≥ 12 seconds in the placebo group. Thus, the proportion of subjects that improved by the smallest measureable change of at least 12 seconds were similar between groups (*p* = 0.88).

### Environmental conditions

The mean environmental conditions of both baseline time-trials were the following: Temperature = 2°C, Dew point = -3°C, Humidity = 78%. For the final time-trial, the mean environmental conditions were the following: Temperature = 10°C, Dew point = 4°C, Humidity = 70%. Thus, overall, there was no statistically significant differences in either temperature, dew point, or humidity measured between groups within any of the three 5-km time trials. However, there was some adjustment for the final 5-km times for a subgroup of seven subjects. Those subjects experienced unseasonably hot/humid weather conditions on their final time trial (Temperature = 22°C, Dew point = 16°C, Humidity = 71%). Based on pace adjustments for temperature and dew points [28], the final times were adjusted down by 2% for only those seven subjects in that final session only.

### Baseline blood parameters

The baseline blood parameters that are listed in the oxidative stress panel provided by Genova diagnostics are presented in Table 2. These data presented are the average baseline values measured twice over a period of one week (7 days). The mean values for both groups were within the reference ranges provided by Genova Diagnostics. There was no difference in any parameter between groups at baseline (rested, fasted, Table 2). The week-to-week coefficient of variation for every blood parameter is presented in Table 3. The coefficient of variation ranged from as low as 5% to a high of 27%.

**Table 2:** Pre and post exercise blood values at baseline and at 88 days following supplementation. The baseline values were averaged over both baseline sessions.

	Reference Range	Pre-exercise	10-minutes post exercise	Change
**Damage**				
Lipid Peroxides (TBARs, μmol/L)	≤ 10			
*Protandim*^®^ *Group*				
Baseline		8.4 (2.1)	8.5 (2.4)	+0.1 (1.7)
88 days post-supplementation		7.4 (2.2)	7.5 (2.6)	+0.1 (1.2)
*Placebo Group*				
Baseline		7.9 (1.9)	8.5 (2.5)	+0.6 (1.9)
88 days post-supplementation		7.7 (3.9)	6.7 (2.5)	-1.0 (4.8)
**Protective Enzymes**				
Superoxide dismutase* (SOD, U/g Hb x 1000)	5.3 to 16.7			
*Protandim*^®^ *Group*		11.5 (3.4)	11.2 (3.5)	-0.3 (1.2)
Baseline		20.2 (8.3)	20.6 (7.4)	+0.4 (3.0)
88 days post-supplementation				
*Placebo Group*		11.7 (3.7)	12.0 (3.5)	+0.3 (1.1)
Baseline		18.7 (6.2)	19.2 (6.7)	+0.5 (3.4)
88 days post-supplementation				
Glutathione Peroxidase (GPX, U/g Hb)*	20 to 38			
*Protandim*^®^ *Group*				
Baseline		27.7 (4.5)	27.3 (4.3)	-0.4 (1.9)
88 days post-supplementation		30.7 (4.4)	31.4 (5.2)	+0.7 (3.4)
*Placebo Group*				
Baseline		28.4 (7.4)	28.6 (7.1)	+0.2 (1.3)
88 days post-supplementation		31.9 (7.8)	31.4 (6.5)	-0.5 (2.8)
**Other**				
Glucose levels (fasted) (mg/dL)*	< 100			
*Protandim*^®^ *Group*				
Baseline		92 (5)	166 (25)	+74 (27)
88 days post-supplementation		91 (5)	158 (46)	+67 (44)
*Placebo Group*				
Baseline		90 (8)	161 (35)	+71 (33)
88 days post-supplementation		91 (9)	166 (43)	+75 (39)
**Reserve**				
Glutathione (GSH) (μmol/L x 10)	≥ 66.9			
*Protandim*^®^ *Group*				
Baseline		103 (13)	103 (11)	0 (9)
88 days post-supplementation		104 (30)	112 (18)	+8 (29)
*Placebo Group*				
Baseline		102 (19)	100 (20)	-2 (8)
88 days post-supplementation		96 (18)	96 (13)	0 (16)
Total Antioxidant Capacity* (TAC, mmol/L)	≥ 0.54			
*Protandim*^®^ *Group*				
Baseline		0.86 (0.07)	0.97 (0.07)	+0.11 (0.06)
88 days post-supplementation		0.80 (0.07)	0.91 (0.09)	+0.11 (0.06)
*Placebo Group*				
Baseline		0.86 (0.06)	0.97 (0.06)	+0.11 (0.04)
88 days post-supplementation		0.80 (0.06)	0.90 (0.07)	+0.10 (0.03)
Cysteine (mg/dL)*	0.61 to 1.16			
*Protandim*^®^ *Group*				
Baseline		0.66 (0.14)	0.67 (0.14)	+0.01 (0.13)
88 days post-supplementation		0.58 (0.13)	0.67 (0.17)	+0.10 (0.14)
*Placebo Group*				
Baseline		0.63 (0.07)	0.69 (0.08)	+0.06 (0.09)
88 days post-supplementation		0.61 (0.14)	0.61 (0.14)	+0.00 (0.10)
Cystine (mg/dL)	1.6 to 1.2			
*Protandim*^®^ *Group*		2.1 (0.4)	2.3 (0.4)	+0.2 (0.2)
Baseline		2.1 (0.4)	2.1 (0.5)	0.0 (0.5)
88 days post-supplementation				
*Placebo Group*				
Baseline		2.2 (0.3)	2.3 (0.2)	0.0 (0.3)
88 days post-supplementation		2.1 (0.3)	2.3 (0.4)	+0.2 (0.2)
Cysteine to Cystine ratio#	0.23 to 0.53			
*Protandim*^®^ *Group*		0.33 (0.12)	0.31 (0.13)	-0.01 (0.08)
Baseline		0.29 (0.12)	0.34 (0.15)	+0.05 (0.08)
88 days post-supplementation				
*Placebo Group*				
Baseline		0.29 (0.07)	0.31 (0.06)	+0.02 (0.08)
88 days post-supplementation		0.29 (0.07)	0.27 (0.07)	-0.02 (0.06)
Sulfate (mg/dL)	3.0 to 5.9			
*Protandim*^®^ *Group*				
Baseline		3.8 (0.5)	4.0 (0.6)	+0.2 (0.6)
88 days post-supplementation		3.9 (0.6)	4.1 (0.8)	+0.2 (0.4)
*Placebo Group*				
Baseline		3.9 (0.9)	4.4 (1.1)	+0.5 (1.0)
88 days post-supplementation		3.8 (0.7)	4.1 (0.8)	+0.3 (0.6)
Cysteine to Sulfate ratio	0.12 to 0.32			
*Protandim*^®^ *Group*		0.18 (0.05)	0.18 (0.05)	0.00 (0.04)
Baseline		0.15 (0.04)	0.17 (0.04)	+0.02 (0.04)
88 days post-supplementation				
*Placebo Group*				
Baseline		0.17 (0.14)	0.17 (0.05)	0.00 (0.04)
88 days post-supplementation		0.16 (0.05)	0.15 (0.04)	-0.01 (0.03)

Mean (SD). There were 16 and 19 subjects in the Protandim Group and Placebo group, respectively, that completed all sessions. This was assessed by a 2 x 4 mixed design ANOVA (2 groups, 4 time-points: pre and post exercise at baseline, and pre and post exercise post-supplementation).

* shows that a main effect of Time was present, even after adjusting for multiple comparisons.

^#^ shows that a Group x Time interaction effect was present (*p* < 0.05).

The reference range was provided to us by Genova Diagnostics.

**Table 3:** The measured week to week coefficient of variation in the blood variables. All blood variables were measured by Genova Diagnostics (n = 38).

Blood Profile components	Week-to-week coefficient of variation (%) (at rest, fasted)	Correlation between session 1 at baseline versus session 2 at baseline (r)	Percent of shared variance between both baseline sessions (r^2^ x 100)
**Damage**			
Lipid peroxides (TBARS, μmol/L)	26%	0.37*	14%
		[0.06 to 0.62]	[0% to 38%]
**Protective enzymes**			
Superoxide Dismutase (SOD, U/g Hb)	24%	0.52*	27%
		[0.24 to 0.72]	[6% to 52%]
Glutathione Peroxidase (GPX, U/g Hb)	15%	0.80*	64%
		[0.64 to 0.89]	[41% to 79%]
**Other**			
Fasting Blood Glucose (mg/dL)	5%	0.64*	41%
		[0.40 to 0.80]	[16% to 64%]
**Reserve**			
Glutathione (GSH) (μmol/L)	16%	0.52*	27%
		[0.24 to 0.72]	[6% to 52%]
Total Antioxidant Capacity (TAC, mmol/L)	7%	0.45*	20%
		[0.15 to 0.67]	[2% to 45%]
Cysteine (mg/dL)	19%	0.24	6%
		[-0.08 to 0.52]	[1% to 27%]
Cystine (mg/dL)	13%	0.62*	38%
		[0.37 to 0.78]	[14% to 61%]
Cysteine to Cystine ratio	27%	0.41*	17%
		[0.11 to 0.65]	[1% to 42%]
Sulfate (mg/dL)	19%	0.40*	16%
		[0.10 to 0.64]	[1% to 41%]
Cysteine to Sulfate ratio	24%	0.37*	14%
		[0.06 to 0.62]	[0% to 37%]

Brackets signify the 95% confidence interval. These samples were from a fasted, rested state.

* The correlation between baseline week 1 and baseline week 2 is statistically significant *p* < 0.05.

#### Chronic effects of supplementation on blood parameters measured at rest

**Fig 2:**
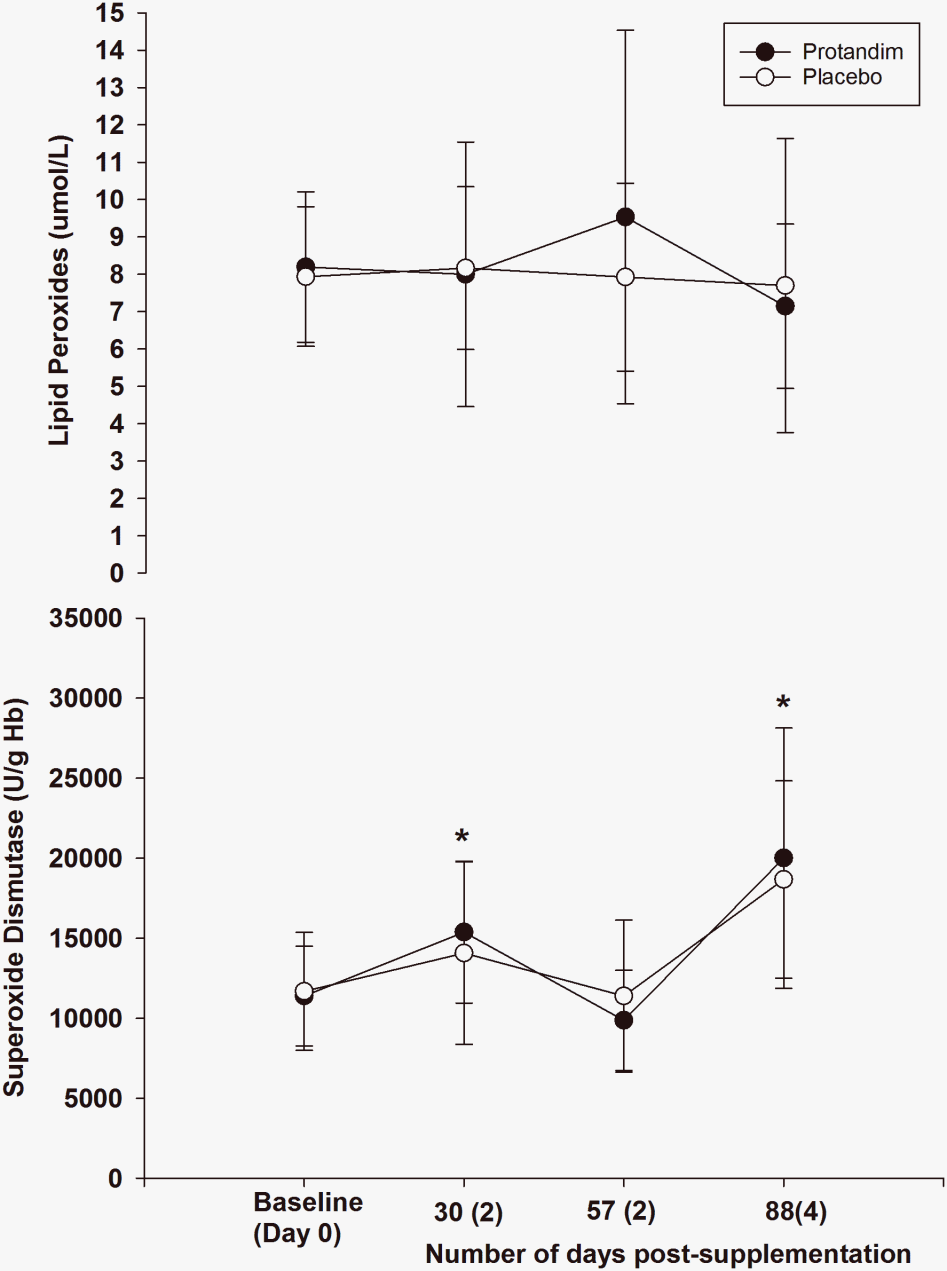
The long term effects of supplementation on lipid peroxides and superoxide dismutase (rest, fasted state). There was no difference between groups for either variable (*p* = 0.74 for lipid peroxides, and *p* = 0.81 for superoxide dismutase). The asterisk* signifies statistical significance for superoxide dismutase at 30 days post-supplementation (*p* = 0.00) and 88 days post-supplementation (*p* = 0.00) compared to the baseline value, after adjustments for multiple comparisons. Mean values represented by circles, error bars represent SD. The x-axis represents the mean (SD) of the number of days post-supplementation.

**Fig 3:**
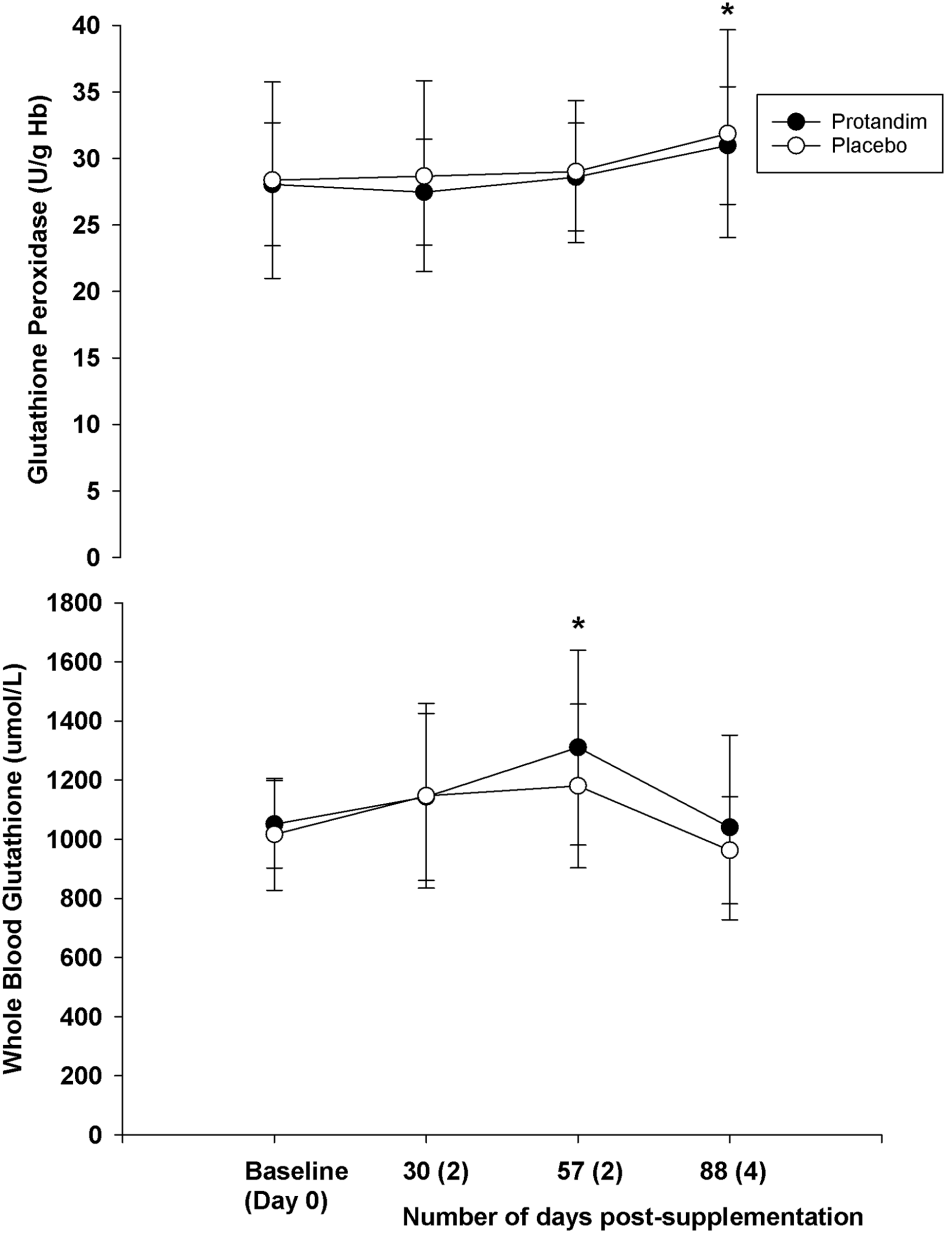
The long terms effects of supplementation on glutathione peroxidase and total glutathione content from whole blood (rest, fasted state). There was no difference between groups for either variable (*p* = 0.66 for glutathione peroxidase, *p* = 0.52 for whole blood glutathione). The asterisk* signifies statistical significance at 57 days post-supplementation (*p* = 0.00) compared to the baseline value after adjustments for multiple comparisons. Mean values represented by circles, error bars represent SD. The x-axis represents the mean (SD) of the number of days post-supplementation.

**Fig 4:**
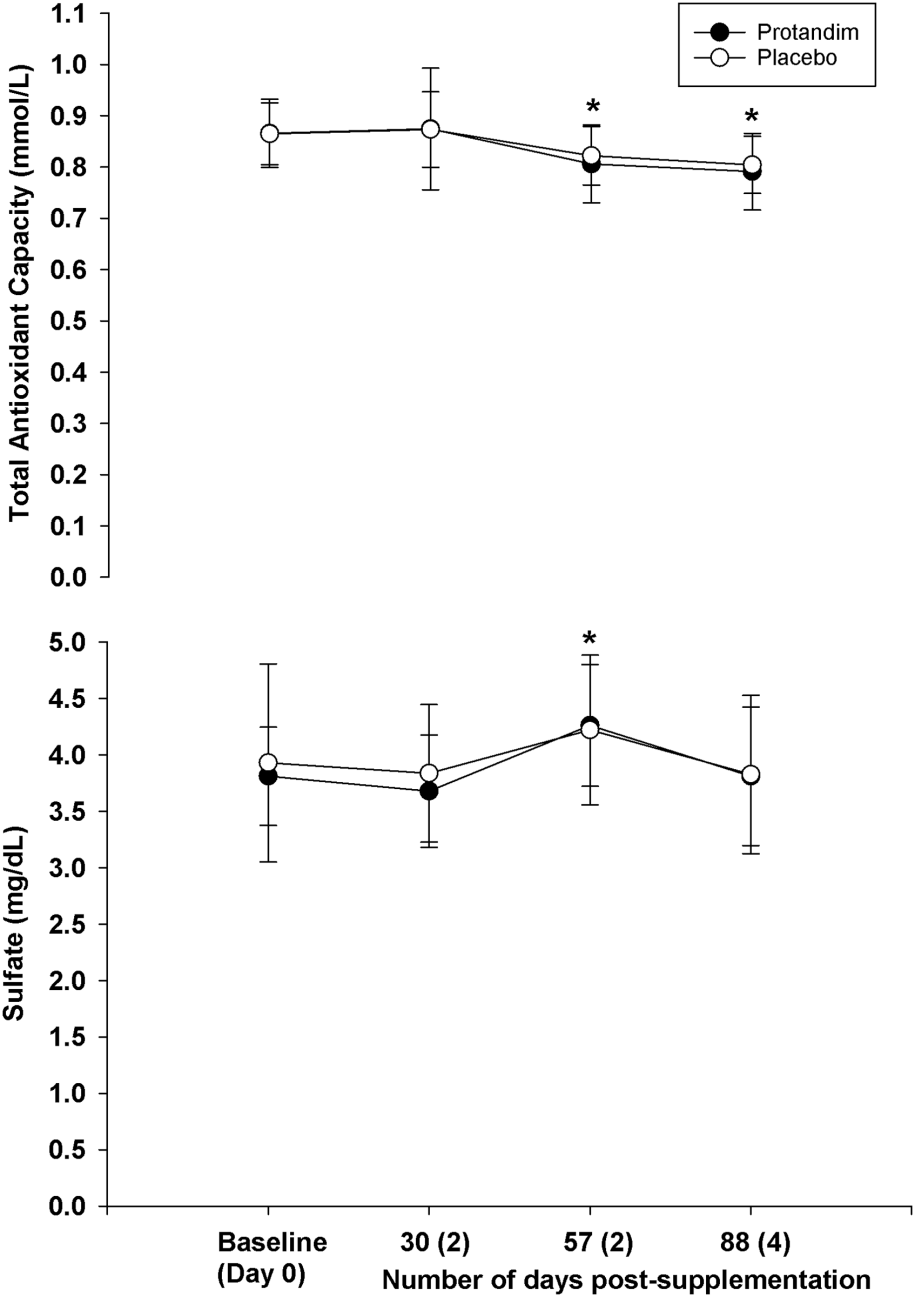
The long terms effects of supplementation on total antioxidant capacity and sulfate (rest, fasted state). There was no difference between groups for either variable (*p* = 0.68 for total antioxidant capacity, *p* = 0.66 for sulfate). The asterisk* signifies statistical significance in the total antioxidant capacity at 57 (*p* = 0.00) and 88 days (*p* = 0.00) post-supplementation compared to the baseline value after adjustments for multiple comparisons. For sulfate, there was a difference at 57 days post-supplementation compared to baseline (*p* = 0.00). Mean values represented by circles, error bars represent SD. The x-axis represents the mean (SD) of the number of days post-supplementation.

**Fig 5:**
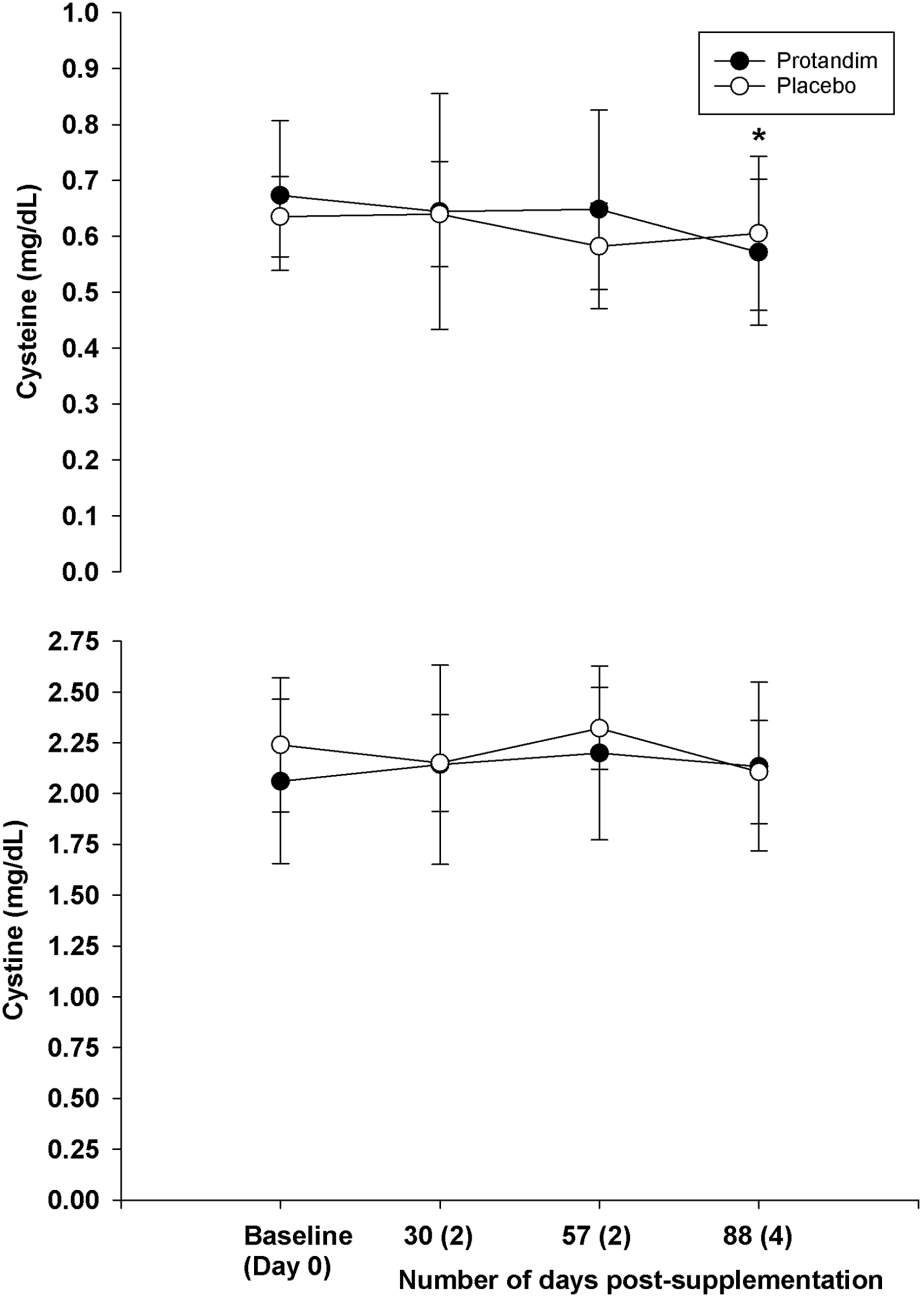
The long terms effects of supplementation on cysteine and cystine (rest, fasted state). There was no difference between groups for either variable (*p* = 0.60 for cysteine, *p* = 0.52 for cystine). For cysteine, there was a difference at 88 days post-supplementation compared to baseline (*p* = 0.013). Mean values represented by circles, error bars represent SD. The x-axis represents the mean (SD) of the number of days post-supplementation.

**Fig 6:**
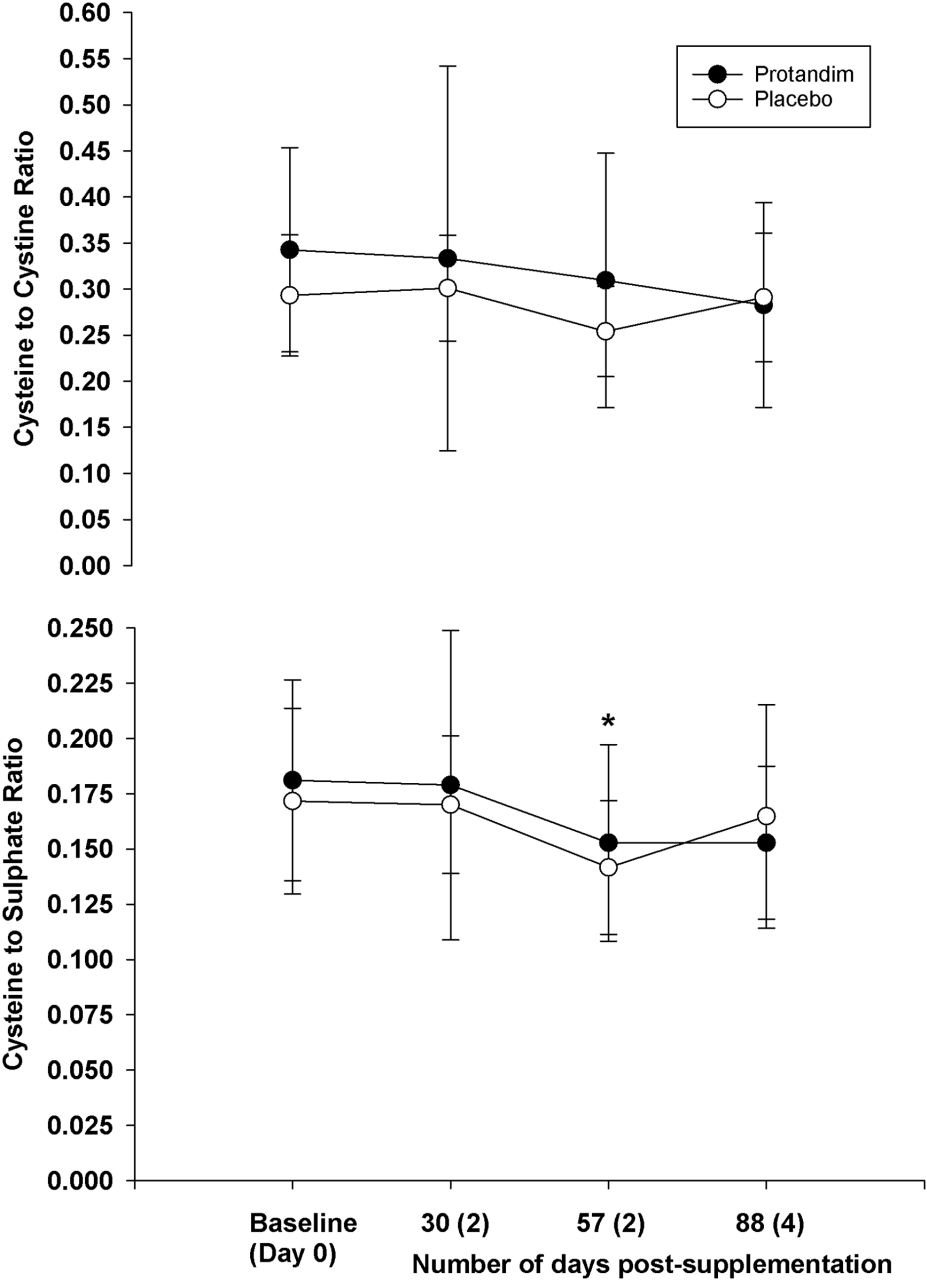
The long terms effects of supplementation on the cysteine to cysteine ratio and the cysteine to sulfate ratio (rest, fasted state). There was no difference between groups for either variable (*p* = 0.30 for the cysteine to cysteine ratio, *p* = 0.69 for the cysteine to sulfate ratio). For the cysteine to sulfate ratio, there was a difference at 57 days post-supplementation compared to baseline (*p* = 0.00). Mean values represented by circles, error bars represent SD.

As a whole, supplementation did not change blood parameters measured at rest compared to placebo (Figs 2–6). However, both groups experiences similar changes across time compared to Day 0 (Baseline).

**Fig 7:**
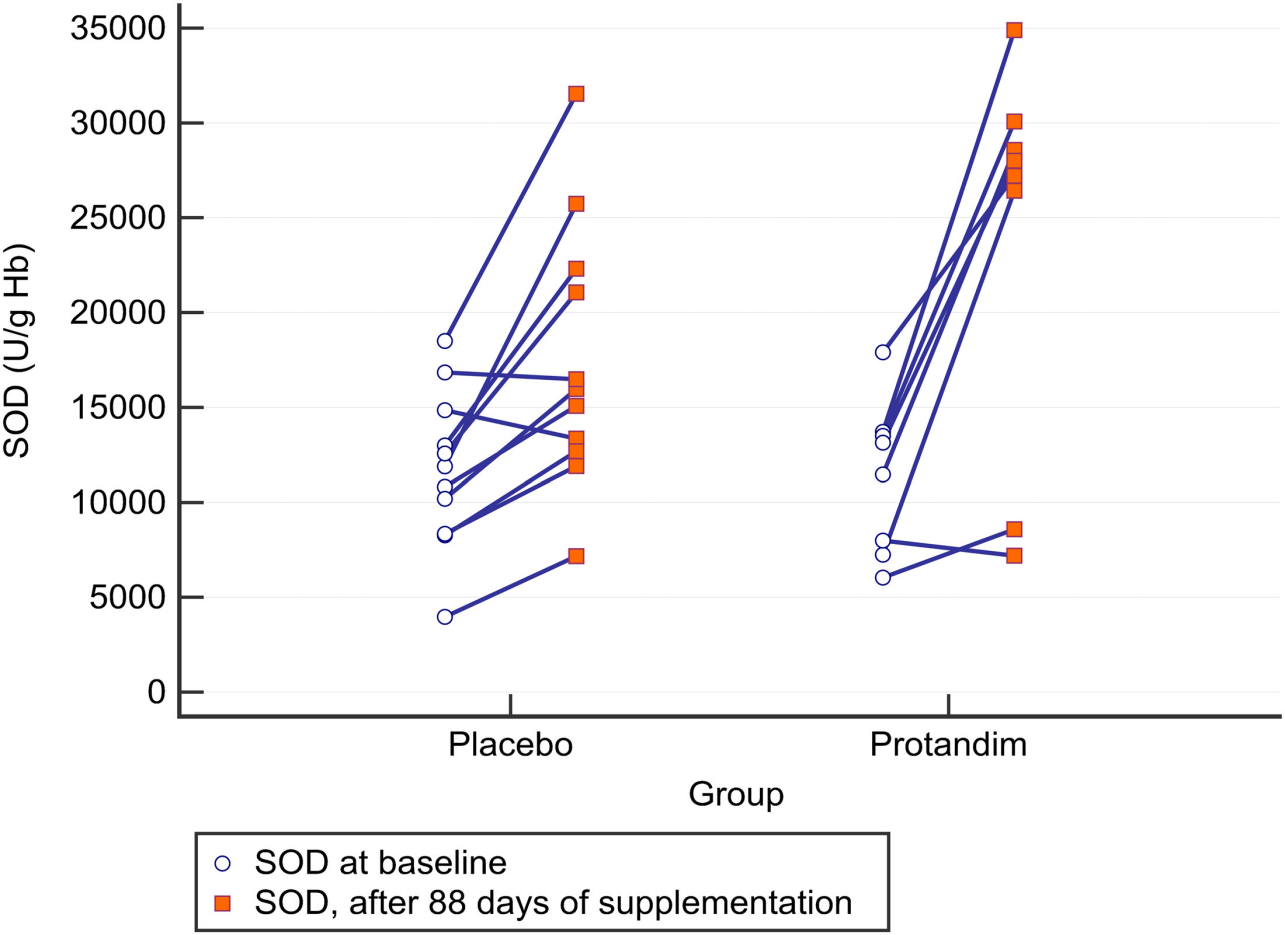
Individual changes in superoxide dismutase (SOD) in runners ≥ 35 years of age (range = 35 to 46 years of age). Those taking Protandim^®^ for 88 days (n = 8) showed a 2-fold higher increase in SOD compared to the 11 runners taking the placebo (Group x Time interaction effect, *p* = 0.038). The effect size for the between group change between Protandim^®^ and placebo was +1.00 (Bias corrected, Hedges) (95% CI of the effect size = 0.03 to 1.96) in favor of Protandim^®^.

Nonetheless, in another *post-hoc* analysis, older subjects (≥ 35 years of age) showed that after 88 days of supplementation, SOD increased by two-fold compared to baseline in the Protandim^®^ group, while the placebo group only experienced a ~50% increase in SOD (Fig 7). The mean increase in SOD in units of enzyme activity per gram of hemoglobin, which is then multiplied by 1000 (U/g Hb x 1000) was significantly larger after 88 days of Protandim^®^ supplementation [mean increase = +12.5 (SD 8.0), 95% CI = +5.8 to +19.2, n = 8] compared to placebo [mean increase = + 5.8 (4.9), 95% = CI +2.5 to +9.1, n = 11] with placebo (*p* = 0.038). The effect size for between group changes was 1.00 (bias corrected, Hedges) in favor of Protandim^®^. No other age related changes were evident GSH, GPX, or TBARS.

### Acute effects of exercise on blood parameters

The 5-km time trials did not affect most blood parameters in the oxidative stress panel. However, TAC increased by ~12%, and blood glucose increased by almost two-fold from pre to 10 minutes post-exercise (Table 2, *p* < 0.0001). Supplementation did not affect these changes.

**Fig 8:**
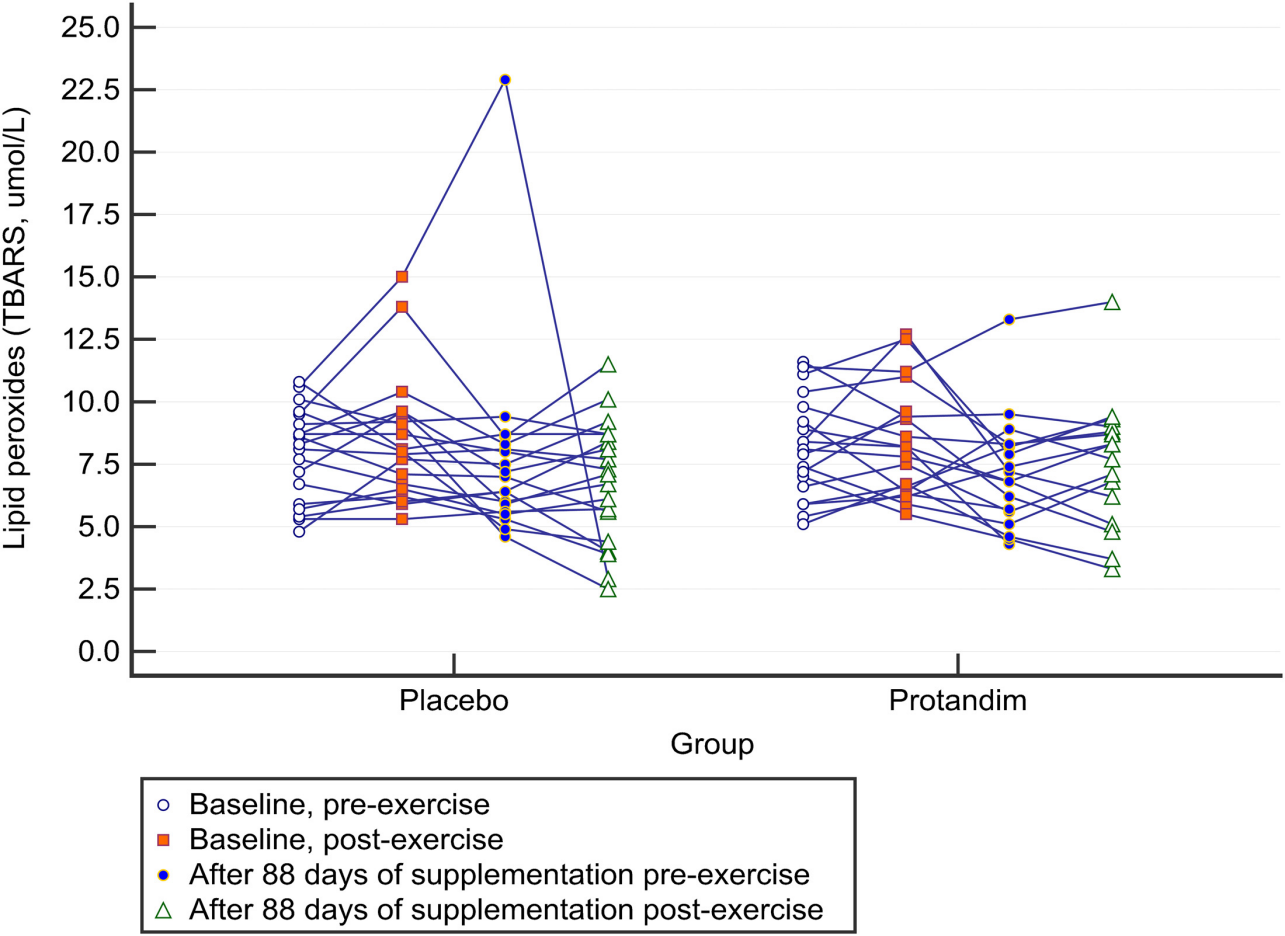
Individual changes in lipid peroxides between groups before the supplementation period and after 88 days of supplementation. The baseline period is an average of both baseline days prior to supplementation. In total, 19 subjects (50%) showed increases in TBARS from pre-race to post-race ranging from 0.1 to 4.4 μmol/L [mean increase = +1.7 (SD 1.4) μmol/L or ~20%]. Supplementation with Protandim^®^ did not lessen the increase in lipid peroxidation compared to placebo.

In total, 19 subjects (50%) showed increases in TBARS from pre-race to post-race ranging from 0.1 to 4.4 μmol/L [mean increase = +1.7 (SD 1.4) μmol/L or ~20%] when both baseline 5-km races were averaged. The individual data points are presented in Fig 8. These subjects were classified as responders to exercise for markers of oxidative stress. This represented 10 out of 19 subjects (~53%) of the subjects in Protandim^®^ group and nine out of 19 subjects (~47%) in the placebo group. However, there were two responders in the Protandim^®^ group had missing post-supplementation data. So, after 88 days of supplementation, 50% of the responders (four out of eight subjects) in the Protandim^®^ group experienced a lower rise in TBARS post-race compared to baseline. In comparison, 89% of the subjects in the placebo group that were responders to exercise for oxidative stress (eight out of nine subjects) experienced a lower rise in TBARS post-race after the supplementation period when compared to baseline. Thus, there was no difference in the proportion of subjects in each group that showed a reduced rise TBARS from exercise after the supplementation period, compared to baseline (50% vs 89%, comparison of proportions, *p* = 0.09). Protandim^®^ was ineffective in reducing post-exercise TBARS for those who demonstrated a rise in TBARS from pre to post race.

### Quality of life analyses

The week-to-week coefficient of variation for the two baseline sessions for QOL raw scores was 4% for Environment and Social Relationships, 5% for Physical Health, and 7% for Psychological Health. There was no significant difference between groups for the any of the four domains throughout the 88 days of supplementation when adjusting for multiple comparisons.

### Signs and symptoms

The total number of events of a given sign/symptom in the Protandim^®^ group over 88 days was 233 episodes (51%), compared to 220 episodes (49%) in the placebo group (Table 4). The number of subjects that experienced at least one event of a given sign/symptom was similar between groups (*p* > 0.05, Fisher’s exact test).

**Table 4:** The total number of participants and events of a given sign / symptom over the supplementation period of 88 days.

Signs/ Symptoms	Protandim^®^ (n = 19)	Placebo (n = 19)	Total number of events from both groups over 88 days
Stomach Ache	5(18)	2(2)	20
Diarrhea	5(10)	2(12)	22
Vomiting	2(2)	(2)	4
Headache	6(40)	3(12)	52
Rash (Hands/Feet)	0	0	0
Gas	1(51)	4(137)	188
Drowsiness	2(3)	4(17)	20
Constipation	2(17)	1(8)	25
Nausea	5(17)	2(7)	24
Dizziness	5(33)	2(5)	38
Insomnia	1(1)	(0)	1
Itching	1(8)	0	8
Joint Pain	2(12)	3(6)	18
Low Blood Sugar	2(18)	(0)	18
Low Blood Pressure	0	0	0
Head cold / congestion	1(1)	0	1
Increased appetite	(0)	(1)	1
Total	(233)	(220)	453

The numbers outside of parentheses represents the number of individuals who had the sign or symptom over the supplementation period. The numbers within the parentheses represent the number of events in each group compared to the summed total of both groups.

All data are provided in S1 Data.

## Discussion

The main objective of this study was to determine whether three months of Protandim^®^ supplementation would improve 5-km running performance in regional class runners and lower serum TBARS. The 5-km track time-trials and serum TBARS measured at rest in a fasted state was not altered in either the placebo or Protandim^®^ groups. A secondary objective was to look at blood antioxidant enzymes (SOD, GPX) at rest and 10 minutes after the completion of the run before and after the supplementation period. Overall, mean SOD and GPX concentration in the blood measured at rest in a fasted state remained unchanged after 90 days of supplementation with Protandim^®^ compared to placebo. However, the SOD concentration at rest showed a larger increase compared to placebo after 88 days of supplementation in those ≥ 35 years of age. Nonetheless, changes in SOD, GPX, and TBARS between rest and 10 minutes post-exercise was similar between groups after 88 days of supplementation compared to baseline.

The data was also examined in terms of proportions and the smallest measureable change. Both Protandim^®^ and placebo groups experienced a similar drop in serum TBARS post-exercise after 90 days of supplementation. Mullins and colleagues found a significant between subject-variability in oxidative stress biomarkers following an intense exercise challenge, suggesting that there may be responders and non-responders to oxidative stress post-exercise [29]. In the current study, 50% of the subjects showed increases in TBARS post-exercise, by an average of 20%. In a *post-hoc* analyses, we examined whether responders to exercise for markers of oxidative damage had lower increases in systemic lipid peroxidation post-race after the 88 day supplementation period. There was no significant difference the proportion of subjects between groups that demonstrated a smaller rise in system lipid peroxidation post-exercise after supplementation period. Thus, 88 days of Protandim^®^ supplementation was unable to mitigate the increase in oxidative damage post-race in those who were responders to an all-out 5 km running race.

A similar proportion of subjects in each group improved 5-km time trial performance by the smallest measureable change of at least 12 seconds. Thus Protandim^®^ was ineffective in improving running performance compared to placebo.

Protandim^®^ marketed by LifeVantage Corporation, is comprised of several phytochemicals which are thought to help the body enhance endogenous antioxidant enzyme production and lower oxidative damage in blood/tissues. It is thought that Protandim^®^ activates the Nrf2 pathway that is integral to the production of several antioxidant enzymes [14].

There has been three human studies to date examining the effects of Protandim^®^ supplementation on oxidative stress. In the first study, Nelson *et al*. [15] demonstrated an age-dependent increase in serum TBARS in normal subjects before supplementation with Protandim^®^. However, supplementation of Protandim^®^ (675 mg/day for 30 days) caused the age-related increase in TBARS to disappear. Subjects who self-reported supplementation with vitamin C and E had significantly higher plasma TBARS. Furthermore, a correlation with age and plasma TBARS was stronger in those that supplemented with Vitamin E and C compared to those that did not supplement with Vitamin E or C, implying that exogenous supplementation of non-enzymatic antioxidants can promote oxidative damage. Lastly, they demonstrated a ~40% decrease in serum TBARS following 30 days supplementation of Protandim^®^ [15]. We saw no such decrease in the present study. Moreover, after 30 days of supplementation, whole blood SOD increased by 8% compared to baseline, and increased by 30% compared to baseline after 120 days [15]. Again, while there was a ~60% increase SOD in the present study after 90 days of supplementation, the placebo group had the same increase (Fig 2). However, in a *post-hoc* analysis in subjects’ ≥ 35 years of age, the increase in SOD after three months of supplementation was larger in the Protandim^®^ group compared to placebo (Fig 7). Caution is needed for this interpretation, as this subset of analysis was not made *a priori*. Performing multiple, unplanned, statistical analyses *after* data is collected is called *p-hacking* and can be inappropriate [30]. To verify that SOD is improved in older subjects after supplementation with Protandim^®^, another study should be performed addressing that specific question. Nelson and colleagues demonstrated that as age increased so did TBARS [15], so the *post-hoc* finding of Protandim^®^ increasing an antioxidant enzyme in the blood like SOD in older individuals could be meaningful, especially when the effect size was large. Even so, Nelson and colleagues did not utilize a placebo controlled, double-blinded design [15], which limits their findings. In the present study, the correlation between TBARS at rest (baseline) and age was 0.274 (*p* = 0.096), but the age range in these runners varied by only 16 years.

Another interesting finding was that 16% of the variance in TBARS at rest (baseline) was accounted for by baseline 5-km race time after controlling for age and baseline training intensity/volume over the previous two weeks (*p* = 0.016, n = 33). Thus, higher circulating TBARS at rest is associated with faster running times. It seems counterintuitive to what one would expect, which would be that those with lower circulating TBARS have faster running times after accounting for age and previous training status. We found the opposite and we have no explanation of why this is so. Again, another study would have to be performed to determine if this is a true association or just a false positive result.

In 2012, a double-blinded, randomized, placebo-controlled trial was published that examined the effect of Protandim^®^ on pulmonary oxidative stress and alveolar permeability in 30 recovering alcoholics [16]. Protandim^®^ was supplemented in 14 subjects at a dose of 1350 mg/day (double the daily dose recommended by the manufacturer) or placebo (in 16 subjects) were administered for 7 days. Relative to placebo-treatment, Protandim^®^ had no significant effects on alveolar epithelial permeability or on TBARS, epithelial growth factor, fibroblast growth factor, interleukin 1β, and interleukin-10 levels in bronchoalveolar lavage fluid. Treatment with placebo, however, produced a significant reduction in plasma levels of TBARS by ~28% [16].

In 2014, an abstract was published in the FASEB journal examining the effects of 30 days of 675 mg/day of Protandim^®^ on serum lipid peroxidation in 13 overweight and/or obese subjects [21]. Ashwagandha is not permitted in dietary supplements in some countries, so black pepper extract (piperine) was substituted for Ashwagandha in this study. Serum TBARS decreased from 6.3 (3.3) to 4.9 (1.7) nmol/mL (*p* < 0.05), or ~22% in the Protandim^®^ (piperine) group. The placebo group, on the other hand, showed no change in serum TBARS post-supplementation [21].

## Limitations

The week-to-week coefficient of variation in several blood parameters was large (e.g. GPX, SOD, and TBARS varied from 15 to 26%) thus, reducing the sensitivity in finding small changes, if there were changes from Protandim^®^. Nevertheless, to our knowledge, this is the only study to have assessed week-to-week variability in these blood parameters at baseline, thus it is unknown whether the variability is due to the technician, machine error, the assay, or from true biological variability. The delay in conducting the assays could have contributed to the variability. Thus, in the future, running the assays immediately after collecting blood samples or having the pre-post samples run on the same assay may reduce variability in subsequent studies.

Some may suggest that serum lipid peroxides was measured too early post-exercise to show any meaningful increases in oxidative damage. There is a range of ideal post-exercise sampling time points used to assess blood markers of oxidative stress. Following the cessation of exercise, each biomarker assessed in the blood can take up to a couple of hours to reach its peak oxidative stress level [7]. For example, the time to highest concentration post exercise for TBARS ranges from 48 to 96 minutes post-exercise, and 96 to 168 minutes for total antioxidant capacity [7]. Thus, in an oxidative stress panel, each parameter has an ideal post-exercise sampling time-point, which varies from parameter to parameter. In the present study, all blood parameters in the oxidative stress panel were measured at the same time post-exercise time-point, which was at ~10 minutes post-exercise, instead of the recommended 48 minutes to 96 minutes post-exercise for serum TBARS [7]. In order to measure serum levels of oxidative damage following an endurance run, multiple blood draws would also need to be performed. However, this would greatly increase cost. Despite this limitation, several studies have assessed oxidative damage within 10 minutes post-exercise and the preponderance of the data demonstrate a measurable increase in serum lipid peroxides post-exercise [3–7]. Even in the study by Michailidis and colleagues, TBARS increased by 41% measured immediately post-exercise from a simple graded exercise test to evolution exhaustion [7]. As a whole, we did not see an increase in TBARS caused by all-out exercise lasting ~20 minutes, even though half of the subjects had a mean increase of 20%.

The validity of serum TBARS in detecting lipid peroxidation has been criticized for a lack of specificity. Serum TBARS is a direct marker of oxidative damage to polyunsaturated fatty acids within cell membranes, otherwise known as lipid peroxidation. But the level of serum TBARS are very general and do not pinpoint where the oxidative damage is occurring in the body. However, many human studies have continued to use serum lipid peroxides as a marker of systemic oxidative damage [3–7] and this study is no different. Nonetheless, serum lipid peroxides do reflect oxidative damage of the liver and heart, at rest, and immediately post-exercise, as demonstrated in rats [24]. Thus, we believe the serum lipid peroxide values measured in our subjects reflect oxidative damage of the heart and liver.

Finally, the primary outcome measures (5-km time trial performance, serum TBARS) and secondary outcome measures (GPX, SOD, other blood markers, and quality of life) as well as the specific research questions described in this manuscript was made to be identical to that of the most recent version to that registered in clinicaltrials.gov. In the original study design, we did not indicate that every blood parameter measured in the oxidative stress panel by Genova Diagnostics would be reported in this paper as part of our analyses. After the fact, however, we decided to report every blood marker provided to us from Genova Diagnostics because we felt that this would make for a more complete paper. So, any blood marker that was analyzed *post-hoc*, like SOD in those ≥ 35 years of age, as well as sulfate, cysteine, cystine, GSH, and TAC should be interpreted with caution.

## Conclusions

In conclusion, this study demonstrated the following:

1. As a whole, regular supplementation of Protandim^®^ (675 mg/day for 88 days) did not improve 5-km time trial performance in regional class runners compared to placebo.
2. Regular supplementation of Protandim^®^ (675 mg/day for 88 days) did not reduce oxidative stress as assessed by serum lipid peroxides (TBARS) in a rested, fasting state compared to placebo.
3. Regular supplementation of Protandim^®^ (675 mg/day for 88 days) increased the antioxidant enzyme SOD by two-fold in subgroup of older subjects (≥ 35 years), compared to only a 50% increase in the placebo group.
4. The 5-km time trials did not acutely increase mean TBARS as a whole, but it did increase by an average of 20% in half of the subjects.
5. Regular supplementation of Protandim^®^ did not reduce the increase in global oxidative damage post-race compared to pre-race in the subgroup of runners who showed increases in TBARS from 5-km running.
6. Regular supplementation of Protandim^®^ did not improve, nor worsen, quality of life in runners.
7. The large variability in the measures of circulating oxidative stress markers and antioxidants warrant the identification of more robust assays, and that the pre-post samples are measured on the same assay.

Future studies are warranted to examine antioxidant enzyme concentrations in blood in older subjects compared to younger subjects after Protandim^®^ supplementation. This would verify that blood SOD is indeed improved with Protandim^®^ supplementation and limit false positive findings.

## Supporting Information

S1 Checklist (PDF)

S1 Data (SAV)

S1 Protocol (PDF)
