# Correction: The Effect of Protandim^®^ Supplementation on Athletic Performance and Oxidative Blood Markers in Runners

**DOI:** 10.1371/journal.pone.0241520

**Published:** 2020-10-23

**Authors:** Seteena L. Ueberschlag, James R. Seay, Alexandra H. Roberts, Pamela C. DeSpirito, Jeremy M. Stith, Rodney J. Folz, Kathleen A. Carter, Edward P. Weiss, Gerald S. Zavorsky

The authors are correcting this article [1] to address queries and errors that were noted following its publication.

Updated versions of Table 2 (S1 File) and Table 4 (S2 File) are included here, as errors in the corresponding tables in [1] were identified following publication.

In the published article [1], when discussing adverse event outcomes (i.e. side effects), the Fisher’s exact test was used to assess the significance of the difference between the number of subjects that experienced at least one event of a given sign or symptom between groups. All p-values were found to be non-significant. When a comparison of proportions between groups was performed (Chi-square, N– 1) [2], the authors found no significant differences between groups in the proportion of subjects experiencing at least one event from any sign or symptom (all p > 0.05) (S2 File). However, due to the limited sample sizes only substantial differences in proportions would be expected to be statistically significant in the analyses of adverse event outcomes; the trial was not designed with enough power to determine whether the proportions of adverse events in the two groups were statistically significant. The analysis of adverse events between groups was not a primary or secondary objective of the study. The adverse events were provided as preliminary data for future studies and for added interest to the reader.

The authors direct readers to S2 File (which includes 3 Tabs) to review the primary data regarding adverse events (side effects) over the 88-day supplementation period. Summary (Tabs 1 and 2) and individual data (Tab 3) are provided here. Of note, one participant in the placebo group accounted for 118 episodes of gas (flatulence), which accounts for a significant amount of the 127 episodes of gas for the Placebo group; 9 different subjects (47%) in the placebo group experienced at least 1 event from any sign/symptom list; and 11 different subjects (56%) in the Protandim^®^ group experienced at least 1 event from any sign/symptom list.

Questions were raised about the selective inclusion of participants aged 35–46 for the analyses in Fig 7 of the original article. As noted in The Funding statement and in the 5^th^ paragraph of the Discussion section, a post-hoc analysis was conducted by LifeVantage Inc. However, these analyses were not included in the original study protocol, and the study did not include enough participants to enable a full assessment of age effects. The authors discussed the issue of *p*-hacking and concerns about unplanned statistical tests in the Discussion (5^th^ paragraph).

As noted in the original article, one of the authors who participated in the randomization of subjects also served as a participant in this study. Here, the authors clarify that this author was blinded to allocation group: GSZ randomized subjects, including himself, to group 1 versus group 2 but did not know which group was experimental versus control. This assignment was determined by the company, which directly allocated the placebo and Protandim supplements coded by numbers and letters. The author was not informed as to the meaning of the codes and became aware of which arm he participated in only when the study was unblinded after the completion of the statistical analyses.

Details as to how subjects were randomized are as follows: In order for the mean 5-km performance time between both groups to be similar at baseline, males and females were ranked separately in randomized blocks of two according to the best 5-km performance time from the first two initial baseline 5-km time trials. Randomized blocks of two were created for males, and then randomized blocks of two were created for females. The males were ranked from fastest to slowest by taking each runner’s best of two baseline 5-km time trials. First and second place males were then placed in a block of two. The third and fourth place males were also in a block of two. And so on. Then a fair coin was flipped. If heads, the first ranked runner in the first pair went to Group 1, otherwise, the second ranked runner in the pair went to Group 1. This was done for each block of two males (each pair of two males). The same was then performed for the women. First and second place females were the first block of two. Then a fair coin was flipped. If heads, the first ranked runner in the first female pair went to Group 1, otherwise, the second ranked runner in the female pair went to Group 1. This continued until all the pairs were allocated into two different groups. This randomization process allowed for the same mean 5-km running times in each group as well as maintaining similar male to female ratios.

The inclusion and exclusion criteria are not reported in detail in the Methods. Interested readers can find this information in the Supporting Information file (S1 Protocol in [1]) or the clinical trial registry (https://clinicaltrials.gov/ct2/show/NCT02172625).

Questions were raised about the inclusion of a participant who dropped out of the study after 2 weeks due to severe side effects (depression). To clarify, this participant’s data were included in Table 4, but the proportion of subjects experiencing depression, or any serious adverse event was not statistically significant between groups (5.3% for Protandim^®^, vs. 0% for placebo).

Primary, individual-level data for the quality of life provided in Supporting Information files with this Correction. The quality of life data was not discussed in detail in the published article [1]. Here, the authors provide a new figure reporting the Quality of Life results following a mixed design analysis of variance (a mixture of between-groups and repeated measures). The mixed design ANOVA was used to compare each domain of the WHOQOL-BREF between groups during the supplementation period (four timepoints). No statistical difference was found at any timepoint between the two groups when Bonferroni-adjusting for multiple comparisons (S3 File shows the figure; S4 File shows the original data).

A linear mixed effects model using restricted maximal likelihood estimation was used to determine if there were any differences between group (Protandim^®^ vs. Placebo), time point (baseline, 30 days, 57 days, and 88 days post-intervention; all at rest), or the two-way interaction between group (Protandim^®^ x placebo) and time point for each blood parameter. A separate linear mixed effects model was developed for each dependent variable (11 blood parameters): lipid peroxides (TBARS), superoxide dismutase (SOD), glutathione peroxidase (GPX), glucose, glutathione (GSH), total antioxidant capacity (TAC), cysteine, cystine, cysteine to cystine ratio, sulfate, cysteine to sulfate ratio. For each model, subject served as the random effect. Group and time point served as categorical fixed effects. An autoregressive moving average repeated measures covariance structure was used for time point. If a significant main effect or two-way interaction was found, Bonferroni-adjusted pairwise comparisons were examined. Alpha was set at 0.05. All statistical analyses were performed in SPSS v25.0.

As was previously mentioned in the original manuscript, the results did not show significant changes in any of the 11 blood parameters with Protandim^®^ supplementation compared to supplementation with placebo when using a repeated measures analysis of variance. When data were analyzed with a linear mixed effects model, they also did not provide evidence that Protandim^®^ altered any of the 11 blood parameters compared to supplementation with placebo (*p* ≥ 0.20 for all 11 blood parameters). There was not a significant Group x Timepoint interaction effect for any of the 11 blood parameters (*p* ≥ 0.18 for all). There were main effects of timepoint for the following blood parameters even after Bonferroni correction: superoxide dismutase (Baseline vs 88 days post-supplementation), glutathione peroxidase (Baseline vs 88 days post-supplementation), glucose (baseline vs 30 days post-supplementation, and baseline vs 57 days post-supplementation), whole blood glutathione (baseline vs 57 days post-supplementation), total antioxidant capacity (baseline vs 57 days post-supplementation, and baseline vs 88 days post-supplementation), sulfate (baseline vs 57 days post-supplementation), and the cysteine to sulfate ratio (baseline vs 57 days post-supplementation) (all p < 0.05).

## Supporting information

S1 File Table 2 Revised. Pre and Post exercise blood values at baseline and at 88 days following supplementation. The baseline values were averaged over both baseline sessions.
(DOCX)

S2 File Table 4 Revised. This Table includes 3 Tabs. Tab 1 provides the comparison of proportions. Tab 2 describes the total number of events between groups. Tab 3 provides the actual individual data. The clinical trials.gov website was used as a reference to determine what was a general adverse event and what was a “serious” adverse event and per their reporting standards, the two are reported separately. Per this reference, depression is considered a serious adverse event and is therefore reported separately. The only serious adverse event reported in any of the subjects was a depression in a single participant, and since only one report existed, it was described in the body of the article (Results section, second paragraph) rather than submitted in the tabular form.
(XLSX)

S3 File A figure depicting the results of the quality of life data. These are the World Health Organization Quality of life (BREF) raw scores. Upper panel A, circles, represent Physical Health scores, triangles represent Social Relationship scores. Lower panel B, circles, represent Psychological Health scores, triangles represent Environment scores. (Black = Protandim®, White = Placebo). Mean values represented by circles and triangles, error bars represent SD. There was no statistical difference between groups or between timepoints after Bonferroni-adjusting for multiple comparisons.
(TIF)

S4 File The quality of life raw data. These are the World Health Organization Quality of life (BREF) raw scores. There are two tabs. One tab in the file provides the definitions of the columns, and the other tab provides the raw data.
(XLSX)
