# Supplementary material for: The Effect of Protandim® Supplementation on Athletic Performance and Oxidative Blood Markers in Runners
Source: PLoS One. 2016 Aug 11;11(8):e0160559. doi: 10.1371/journal.pone.0160559 (PMC4981460; doi:10.1371/journal.pone.0160559)
Supplement: S1 Protocol — (PDF) [file pone.0160559.s003.pdf]

The effects of 90 day supplementation of Protandim® on markers of oxidative stress, athletic performance, and recovery

Gerald S. Zavorsky PhD, FACSM

Introduction

Oxidative stress occurs when the production of reactive oxygen species (ROS) outweighs the body's ability to remove them. Reactive oxygen species is a general term for molecular oxygen-derived molecules that are converted easily to reactive species, some of these species are free radicals[1]. A free radical (unpaired electron in the molecular orbital) can oxidize a biomolecule such as a nucleic acid, a protein, or a lipid, which then alters genetic information, it can denature the protein, inactivate an enzyme, and thus disturb a bio-membrane[1]. Thus, development of ROS causes oxidative stress, which caused by oxidation due to a disturbance in the oxidant-antioxidant balance[1].

Main cellular antioxidant defenses include superoxide dismutase (SOD) and glutathione peroxidase (GPX)[2]. Superoxide dismutase provides the first line of enzymatic defense against intracellular free radical production by removing a one-electron dismutation of oxygen (O_2^-) to hydrogen peroxide (H_2O_2)[2]. Glutathione peroxidase catalyzes the reduction of H_2O_2 and organic hydroperoxide to water and alcohol, respectively, using GSH as the electron donor[2]. Glutathione serves as a substrate for GPX to remove hydrogen and organic peroxides (e.g. lipid peroxides)[2]. By donating a pair of hydrogen ions, GSH is oxidized to oxidized glutathione (GSSH). Reduced glutathione measured in the blood can adequately reflect the redox status of skeletal muscle and heart[3].

Acute exercise training can promote oxidative stress (**Table 1**). One measurement of oxidative stress is plasma thiobarbituric acid-reactive substances (TBARS). TBARS is a strong marker of lipid peroxidation that reflects oxidative damage to DNA[4]. From **Table 1** it can be seen that acute strenuous exercise increases TBARs by 21 to 45%. TBARS measured in the blood can describe the redox status of the heart and liver[3].

Physical overtraining can also increase oxidative stress. It has been shown that three weeks of six days per week of high intensity resistance training increased TBARS by 56% while reducing reduced GSH by 31%, and total antioxidant capacity (TAC) by 20%[5].

Regular endurance training can increase enzymatic SOD and GPX by 25 to 35% in muscle[6] and 45% in blood[5]. Thus, proposer exercise training (not too much, not too little) can increase antioxidant defenses.

Nutrition supplementation with antioxidants have been discussed as a way to further enhance overall well-being of athletes, faster recovery, and minimization of injury time, and overall improved performance[7]. One study used Resurgex Plus (which includes 500 U of an oral form of SOD and 1.5 grams of fruit polyphenols) in 24 college soccer players to see if the supplement decreased muscle damage and reduced oxidative stress[8]. After 20 days, 2 x per day supplementation, performance was not improved with this supplement compared to placebo group. However, one marker of oxidative stress, lipid hydroperoxide, seemed to be blunted post-

exercise compared to pre exercise after supplementation with Resurgex Plus (2.3 fold increase in lipid hydroperoxide before supplementation, to 1.9 fold increase post-supplementation, p = 0.067). The rise in creatine kinase (a marker of muscle damage), post-workout compared to pre-workout was smaller with the supplementation (p = 0.044)[7].

More work in the rea of antioxidant supplementation on athletes needs to be done. Another nutrition supplement company, LifeVantage (http://www.lifevantage.com/ has a product named Protandim® that reduces oxidative stress (http://www.lifevantage.com/products/protandim/). In one study, twelve subjects, 20 to 78 years of age ingested 675 grams (one capsule daily) of Protandim® (Bacopa extract 150 mg; milk thistle 225mg, ashwagandha 150 mg, green tea 75 mg, turmeric 75 mg) for 120 days. Since the life of a red blood cell is 120 days, erythrocyte SOD was measured after 120 days of Protandim® supplementation. It would therefore be expected that after 120 days, 100% of all erythrocytes have turned over, reflecting new steady state levels of SOD. After 30 days of supplementation, SOD increased by 8% compared to baseline, and increased by 30% compared to baseline after 120 days[9]. In another group of subjects (n = 4), the amount of lipid peroxidation (TBARS) decreased by 50% after 30 days of supplementation of 338 g/day Protandim®, which was not reduced further after 120 days[9]. Thus, a major reduction in oxidative damage, as expressed by TBARS, is noticeable after five days of supplementation, and peaks after 30 days of supplementation[9], while erythrocyte SOD is at its highest levels after 120 days supplementation[9].

Purpose

In the past ten years there has been several studies published using the nutritional supplement Protandim® [9-16], but, to my knowledge only three were human studies[9,10,16]. About 54 of these subjects supplemented with Protandim®. No side-effects were reported in these studies. No study to date has examined the effects of Protandim® on oxidative stress and endurance performance, although a similar study has been done using another supplement[8]. Thus, the purpose of this study is to examine the effect of ~90 day Protandim® supplementation on 5-km running performance on acute and long term oxidative damage as assessed by blood markers (**Table 2**). Secondarily, another purpose of this study is to examine the effect of Protandim® supplementation on measures of measures of quality of life. The experiment will be double-blind, placebo controlled. The study will address four specific research questions:

1. Does regular supplementation of Protandim® (675 mg/day for 90 days) reduce oxidative damage at rest as assessed by lipid peroxides (TBARS) in runners? Hypothesis: Based on the data by Nelson et al. (2006)[9], oxidative damaged will be reduced by 40% (effect size = 4.8)

2. Does regular supplementation of Protandim® (675 mg/day for 90 days) in runners reduce the increase in oxidative damage post-race compared to pre-race as assessed by lipid peroxides (TBARS)? Hypothesis: Based on the data by Kyparos et al. (2009)[17], we expect a 45% increase in TBARS post-exercise compared to pre-exercise (effect size of 3.6). However, we expect that those that are on Protandim® for 90 days will only have

31% increase in TBARS post-race (effect size = 2.5) [based on changes in pre-post lipid peroxides from Arent et al. (2010)[8]]

3. Does regular supplementation of Protandim® (675 mg/day for 90 days) improve 5-km running times? Hypothesis: Protandim® will improve 5-km running time by 0.5 min (SD 1-min). This is equivalent to an effect size of 0.5. The placebo group will have no change in performance.

4. Does regular supplementation of Protandim® (675 mg/day for 90 days) improve either the physical or mental component of quality of life according to the SF-36 questionnaire[18-20] or the physical and psychological component of the WHOQOL-BREF[21]? Hypothesis: There will be a statistical improvement in the quality of life post-Protandim® supplementation which a small effect size improvement of 0.33.

Procedures

A total of forty apparently healthy community runners 18 to 55 yrs of age will be recruited to participate in this study that will require approximately six total visits to the University of Louisville lab at Crawford gym, which includes three sessions at the running track (or cardio-path) at University of Louisville (**Table 3**).

The inclusion criteria will be the following:

a) Healthy, non-pregnant subjects between 18 and 55 years of age. Apparently healthy is classified as A-1, A-2, or A-3 according to the American Heart Association[22].

b) Subjects have to achieve a classification of "local class" based on age-graded time[23]. The age-graded time is the finish time adjusted to that of an open division participant using a factor for age and gender. Thus, the times for women and older participants are adjusted downward, while the times for most open division participants (such as 25-year-old men) remain the same. For example, a 55 year old woman has to run 29 minutes 45 seconds or better to be in the study[23]. For a man the same age, the equivalent time would be 25 minutes 2 seconds[23]. This is approximately 60% of the speed of the current world record time for that age[23] (See **Table 4**).

c) Subjects who will abstain from taking any nutritional supplements for the duration of the study, including vitamins and mineral supplements (Exception, ferrous sulfate, elemental iron, Vitamin D, Calcium). Subjects will also abstain from taking any over the counter products (herbals, melatonin, St. John’s Wort, etc…) for the duration of the study.

The exclusion criteria will be the following:

a) Under 18 and over 55 years of age; those who are not apparently healthy is classified as A-1, A-2, or A-3 according to the American Heart Association.

b) Subjects who are not able to run 5-km in the time required for their age and gender (**See Table 4**).

c) Subjects that will continue to take nutritional supplements, including over the counter products, for the duration of the study, including vitamins and mineral supplements (exception: Ferrous sulfate, Elemental iron, Vitamin D, Calcium).

d) Subjects that are taking prescription medications with the exception of birth control.

e) Known allergy or sensitivity to milk thistle, Bacopa monnieri, Ashwagandha, turmeric (or ginger), tea, its parts, caffeine, tannins, or members of the Theaceae family.

A total of about 24 teaspoons of blood (~120 mL, or about 2 to 3% of total blood volume) will be withdrawn from a peripheral vein for the duration of the study (about 105 days). Runners will be recruited from running clubs across the local community. Recruitment flyers will be posted in running stores all over the city and through the daily University of Louisville e-mail blasts, Facebook and other social media. These participants will be randomized into two groups (Protandim®, Placebo). The groups will be sex and performance matched after the initial 5-km race is run.

Each subject will undergo six testing sessions as outline in **Table 3**.

Session 1 (Initial Screening Day)

Subjects will sign a consent form and then they will complete a physical activity readiness questionnaire (PARQ)[24] to clear them for physical activity. Age, weight, and height will be recorded. Subjects will be asked to list all the nutritional supplements they are taking, including multivitamins. They will also be asked to list the 5-km personal best time, and when that was done. They will also report most recent 5-km time and when that was achieved. Then, they will be asked to refrain from taking any multivitamins or nutritional supplements for the duration of the study due to the previous evidence that Vitamin E and C supplementation affects plasma TBARS[9]. The exception will be that subjects are allowed to take Ferrous sulfate, elemental iron, Vitamin D, and Calcium Supplementation if they were taking it before to help counteract previous low hemoglobin levels. The subjects will record what they eat during the 24 hr prior to the test day, and then ask them to eat the same thing and do same activity before all follow-up tests. Then a training diary will be given to them to fill in for the duration of the study. The training diary will include intensity, training duration, and mileage per week. After, they will be given six quality of life questionnaires [World Health Organization's WHOQOL-BREF quality of life assessment[21]] to fill out at home (one each for Session 1-6)]. A pregnancy test will also be conducted.

Session 2 (First Baseline, About 15 days after Session 1)

Subjects will be fasted in the morning when they will arrive at University of Louisville for their pre-exercise blood sampling. They will bring their 24-hr dietary recall with them and their quality of life forms. Approximately three teaspoons of blood will be withdrawn 30-60 minutes

prior to exercise for analysis of several blood parameters (the analytes are listed in **Table 3**). Then subject will be required to partake in a 5-km time trial at the University of Louisville track or on the University of Louisville Cardio-path. Time trials have greater logical validity compared to time-to-exhaustion tests[25], so that is why time-trials are selected as the performance measure.

About 10-minutes post-exercise, another three teaspoons of blood will be withdrawn from each subject. Only after their post-exercise blood draw will the subjects be able to eat.

Based on the performance of the 5-km time trials, males and females will be ranked separately. This will be done in randomized blocks of two. Randomized blocks of two for males, and then randomized blocks of two for females. For example, 20 males will be ranked from the 5-km race from 1st (fastest runner) to 20th (slowest runner). First and second place males will be a block of two. Third and fourth place males will be a block of two. And so on. Then a fair coin will be flipped. If heads, the first ranked runner in the first pair will go to Group 1, otherwise, the second ranked runner in the pair will go to Group 1. This done for each block of two males (each pair of two males). The same is then performed for the 20 women. Twenty females will be ranked from first to 20th. First and second place females will be the first block of two. Third and fourth place females will be the second block of two. Then a fair coin will be flipped. If heads, the first ranked runner in the first female pair will go to Group 1, otherwise, the second ranked runner in the female pair will go to Group 1. And so one. This randomization should allow for the same mean 5-km running times in each group as well as maintaining similar male to female ratios.

Session 3 (Second baseline, about 7 days after Session 2)

Subjects will be fasted in the morning when they will arrive at University of Louisville for their pre-exercise blood sampling. They will bring their 24-hr dietary recall with them and their quality of life forms. Approximately three teaspoons of blood will be withdrawn 30-60 minutes prior to exercise for analysis of several blood parameters (the analytes are listed in **Table 3**). Then subject will be required to partake in a 5-km time trial at the University of Louisville track or on the University of Louisville Cardio-path.

About 10-minutes post-exercise, another three teaspoons of blood will be withdrawn from each subject. Only after their post-exercise blood draw will the subjects be able to eat.

Depending on the Group, they will be given approximately about a 90 day supply of Protandim® or Placebo (corn starch and food coloring). The study is double blinded so neither the researchers nor the subjects will know which group is the Protandim® group and which group is the Placebo group. They will be asked to ingest 1 pill per day, ideally with breakfast (675 mg per day, 1 pill per day). The subjects will be given the complete supply of pills during this session, right after the second blood sample is taken. The subjects will be classified as Group 1 or Group 2 based on their five kilometer results from the first session. The pill bottles will be labeled Group 1 or Group 2. The pill bottles will be dispensed by the research team according to group number after their second blood sample. Subjects will be ask to record the daily intake of the pills in their training diary. Once the study is completed, subjects will be asked to return the pill bottles to the research staff. That way, the researchers are able to account for any missed days.

Session 4 (About 23 Days after Session 3)

Subjects will be fasted in the morning when they will arrive at University of Louisville for their pre-exercise blood sampling. They will bring their 24-hr dietary recall with them and their quality of life forms. Approximately three teaspoons of blood will be withdrawn. There will be no 5-km running race performed in this session. A pregnancy test will be conducted in the females.

Session 5 (about 30 Days after Session 4)

Subjects will be fasted in the morning when they will arrive at University of Louisville for their pre-exercise blood sampling. They will bring their 24-hr dietary recall with them and their quality of life form. Approximately three teaspoons of blood will be withdrawn. There will be no running race performed in this session. A pregnancy test will also be conducted in the females.

Session 6 (About 30 days after Session 5)

Subjects will be fasted in the morning when they will arrive at University of Louisville for their pre-exercise blood sampling. They will bring their 24-hr dietary recall with them and their quality of life forms. They will also bring any unused pills for proper documentation. A pregnancy test will be conducted in the females. Approximately three teaspoons of blood will be withdrawn 30-60 minutes prior to exercise for analysis of several blood parameters (the analytes are listed in **Table 3**). A pregnancy test will then be conducted. After, subjects will be required to partake in a 5-km time trial at the University of Louisville track or on the University of Louisville Cardio-path.

About 10-minutes post-exercise, another three teaspoons of blood will be withdrawn from each subject. Only after their post-exercise blood draw will the subjects be able to eat.

Things to Consider

The between subject-variability in oxidative stress biomarkers is large[26] and suggests that there may be responders and non-responders to an oxidative stress challenge[26]. Individuals who have large increases in oxidative stress (i.e. responders) could be reflecting an inability to regulate redox homeostasis and warrant additional support[26] (i.e. with antioxidant supplementation with Protandim®). It is also necessary to obtain week-to-week and month-to-month coefficient of variation in markers of oxidative stress so that meaningful changes in these markers can be evaluated. Thus, the placebo group will be allow us to obtain the week-to-week and month to month coefficient of variation. As well, for the same reason, it is necessary to obtain week-to-week and month-to-month variation in running performance, thus, the placebo group will allow for the development of the coefficient of variation in performance.

Measuring dietary data is troublesome. First, questionnaires don't give details about antioxidant intake - rather they give outcomes like fruit and vegetable intake, etc. More in depth measures wouldn't be worth the trouble. Thus, having subjects record what they eat (and their physical activity) during the 24 hr prior to the test day, and then ask them to eat the same thing and do same activity before the follow up tests at seven days and 90 days will be required.

Environmental conditions will be recorded on the days of the three time trials (wind, temperature, % humidity, barometric pressure).

Once all the data is collected and analyzed LifeVantage will reveal which group was which.

Statistical Analyses

Once all the data is collected and analyzed, the LifeVantage will reveal which group was which. The main dependent variables measured will be 5-km finishing time, TBARS, SOD, and both the physical and mental/psychological component of the quality of life questionnaire. Based on a 5-km time improvement of 2.5% or about 30 seconds (SD = 1 minute) with Protandim®, and no improvement in the placebo group, about 33 runners in total will be needed (Effect size for ANOVA $f = 0.24$, statistical power = 80%, alpha error probability = 5%, Correlation amongst repeated measures = 0.60, F-test Family, ANOVA repeated measures, between-within interaction, G*Power 3.1.2, Universität Kiel, Germany). Accounting for about an 18% attrition rate (7 subjects), a total of 40 subjects will be recruited (20 per group).

Additional thoughts:

1. Each research subject will have 15 mL of blood withdrawn from a peripheral vein at each sampling time-point. There will be eight blood withdrawals, so that is about 120 mL for the whole study (equivalent to 24 teaspoons). Subjects will be required to fill out the quality of life questionnaire six times, at 15 minutes per questionnaire (90 minutes in total). They will be required to fill in a daily training diary for about 105 days (10 minutes per day for 105 days = 1050 minutes). They will also be filling in a dietary recall form of what they ate 24 hrs before blood sampling (Five forms in total = 50 minutes in total). In addition, the subjects will be required to run three 5-km races (a total of about 60 minutes of running for the whole study, plus 60-minutes of warm-up and warm-down). In addition, subjects will be required to spend about 30-minutes in session 1 in the lab for the initial screening day. As such, that is about 22.3 hours in total for the full duration of the study. Thus, $300 per subject is adequate for their time devoted to the study. ($12,000 in stipends).

Possible Risks of Ingesting Protandim®

We do not expect there to be any side effects for the typical Protandim® consumer. However, according to the Protandim® website (http://www.protandim.com/faqs), some individuals have natural allergic responses to one or another of the ingredients, just as some people are allergic to pine pollen or penicillin. These allergic responses to Protandim® generally appear as gastrointestinal disturbances (i.e., stomach ache, diarrhea, vomiting) or sometimes as a headache or rash on the hands or feet. The symptoms disappear if Protandim® is discontinued.

However, according to the Natural Standard Database (https://naturalmedicines.therapeuticresearch.com/), each botanical has been shown to have rare side effects. They are listed on the next page grouped with each botanical.

MILK THISTLE: According to the Natural Standard Database, the 225 mg dose of Milk thistle that is in each Protandim® pill is likely safe. However, there are some rare side effects that may occur. Milk thistle may cause allergic skin reactions, bloating, blood clots, collapse, constipation, decreased platelets, diarrhea, eczema, elevated liver enzymes, fever, gas, giddiness, headache, heart attack, heartburn, high bilirubin (a toxic substance) in the blood, hives, impotence, increased creatinine, increased lactate dehydrogenase level, infection, insomnia, irritability, itching, joint pain, liver damage, loss of appetite, nausea, non-specific muscle and joint effects, pounding heart, rash, severe allergic reactions, sexual dysfunction, stomach distress or pain, skin pigment lightening, skin reactions, sweating, taste changes, tremor, vomiting, and weakness. Milk thistle may lower blood sugar levels.

BACOPA EXTRACT: According to the Natural Standard Database, the 150 mg dose of Bacopa extract that is in each Protandim® pill is likely safe. However, there are some rare side effects that may occur include nausea, dry mouth, thirst, and fatigue. Bacopa has been reported to cause palpitations (irregular heartbeats).

ASHWAGANDHA: According to the Natural Standard Database, the 150 mg dose of ashwagandha that is in each Protandim® pill is likely safe. However, there are some rare side effects that may occur. Side effects of ashwagandha include the risk of bleeding, promote low blood sugar levels, promote low blood pressure, and may cause drowsiness.

TURMERIC: According to the Natural Standard Database, the 75 mg dose of turmeric that is in each Protandim® pill is likely safe. However, there are some rare side effects that may occur. Side effects of turmeric include an altered heartbeat, an increase in blood volume, an increase in menstruation period, an increase in urine flow, changes in skin color, changes in cholesterol (decrease "bad" cholesterol), changes in immune function, changes in thyroid function, common cold, constipation, decrease in fertility (decrease sperm counts), decrease in iron absorption, delusion, diarrhea, gallbladder contraction, gas, giddiness, gout pain, hair loss (high doses), heartburn, hormone changes, inflammation of stomach and intestine lining, irritated or itchy skin, kidney stones, liver cell toxicity, liver function changes, mild fever, oxidative stress, nausea, rash, skin hardness, skin papules, skin redness, stomach fullness and pain, stomach ulcers, throat infection, transient complete atrioventricular block (a heart measurement), uterine contractions, vertigo, weight loss, worsening of arthritis symptoms, and yellow or hard stools. Turmeric may increase the risk of bleeding. Turmeric may lower blood sugar levels and blood pressure.

GREEN TEA: According to the Natural Standard Database, the 75 mg dose of green tea that is in each Protandim® pill is likely safe. However, there are some rare side effects that may occur. Side effects include affecting blood sugar and blood pressure levels.

Table 1. Acute effects of exercise on blood markers of oxidative stress

	Pre	Post	Change	Effect size
**Kyparos et al. (2009)[17]<br>19 rowers (19 yrs old) performed a 7 minute row<br>Same data as Kyparos et al. (2012)[27]**				
TBARS (μmol/L)	4.0 (0.5)	5.8 (0.5)	+ 1.8* (45%)	3.6
TAC (mmol/L)	0.68 (0.01)	0.74 (0.01)	+0.06* (9%)	6
Reduced GSH (μmol/L)	520 (30)	500 (30)	-20 (-4%) NS	--
Catalase (mmol/L per min)	14 (1.4)	28.7 (3.8)	+14.7 (105%)*	10.5
**Kabaskalis et al. (2011)[28]<br>5 swimmers (29 yrs old) swam for 19.4 hrs**				
TBARS (μmol/L)	8.2 (2.5)	9.9 (2.4)	+1.7 (21%)*	0.7
TAC (mmol DPPH/L serum)	1.0 (0.1)	1.0 (0.2)	NS	--
**Mullins et al. (2013)[26]<br>30 subjects (22 yrs old) performed a graded exercise test to exhaustion for 13 minutes**				
GPX (U/L)	81.3 (11.6)	87.7 (10.4)	+6.4 (+8%)*	0.55
TAC (mmol/L)	2.0 (0.1)	2.0 (0.1)	NS	--

All measurements post-exercise were taken 10 minutes or less post-exercise.

Table 2. Parameters measured from blood. Titled: Oxidative Stress 2.0 (Blood). From Genova Diagnostics: http://www.gdx.net/product/10027

Protection	Method of analysis	Reference range
**Glutathione (GSH)**	Colorimetric assay	≥ 669 µmol/L
**Total Antioxidant Capacity (TAC)**	Enzymatic reaction assay	≥ 0.54 mmol/L
**Cysteine (Cys-SH)**	Colorimetric assay	0.61 to 1.16 mg/dL
**Sulfate**	Turbidimetric assay	3.0 to 5.9 mg/dL
**Cysteine to Sulfate ratio**	-	0.12 to 0.32
**Cystine (Cys-S-S-Cys)**	Colorimetric assay	1.60 to 3.22 mg/dL
**Cysteine/Cystine Ratio**	-	0.23 to 0.53
**Enzymes**		
**Glutathione Peroxidase (GPX)**	Enzymatic reaction assay	20 to 38 U/g Hb
**Superoxide Dismutase (SOD)**	Colorimetric assay	5275 to 1662 U/g Hb
**Damage**		
**Lipid Peroxides**	Thiobarbituric acid-reacting substances (TBARS) assay	< 10µmol/L

Table 3. Proposed timeline of study

	Session 1<br><br>Initial Screening Day<br>(15 days before study begins)	Session 2<br>(Day 1)<br><br>First baseline 5km race	Session 3<br>(Day 7)<br><br>Second baseline 5km race	Session 4<br>(Day 30)	Session 5<br>(Day 60)	Session 6<br>(Day 90)<br><br>Final 5km race
**Protandim® group or Placebo group (n = 20 per group)**	-Consent form signed<br><br>-PARQ signed<br><br>-Age, height weight obtained<br><br>- Obtain information of current supplement use. Ask to restrict supplement use for duration of study<br><br>-Current training diary to be filled<br><br>- Give QOL form to them to fill out in lab<br><br>- Give QOL form to fill out at home<br><br>- Give 5 forms to list what subjects ate 24 prior<br><br>-urine test to rule out pregnancy (women)	-Collect QOL form<br><br>- Collect form that lists what subjects ate 24 hrs prior<br><br>-weight obtained<br><br>-Fasted blood sampling 1 hr before race<br><br>-5km race<br><br>-Post-exercise blood sampling (10 min post)<br><br>- Give QOL form to them to fill out	-Collect QOL form<br><br>- Collect form that lists what subjects ate 24 hrs prior<br><br>-weight obtained<br><br>-Fasted blood sampling 1 hr before race<br><br>-5km race<br><br>-Post-exercise blood sampling (10 min post)<br><br>-Provide this group with 675 g/ day Protandim® for 90 days. Or 675 g/ day placebo for 90 days<br><br>- Give QOL form to them to fill out	-Collect QOL form<br><br>- Collect form that lists what subjects ate 24 hrs prior<br><br>-urine test to rule out pregnancy (women)<br><br>-weight obtained<br><br>-Fasted Blood Sample<br><br>- Give QOL form to them to fill out	-Collect QOL form<br><br>- Collect form that lists what subjects ate 24 hrs prior<br><br>-urine test to rule out pregnancy (women)<br><br>-weight obtained<br><br>-Fasted Blood Sample<br><br>- Give QOL form to them to fill out	-Collect QOL form<br><br>- Collect form that lists what subjects ate 24 hrs prior<br><br>-Collect training diary<br><br>-Collect unused pills for measure<br><br>-urine test to rule out pregnancy (women)<br><br>-weight obtained<br><br>-Fasted blood sampling 1 hr before race<br><br>-5km race<br><br>-Post-exercise blood sampling (10 min post)

*See Table 2 for a list of the blood biomarkers collected

Table 4. The slowest 5-km run time that a subject needs to run to be allowed to participate in the study.

Age	Men	Women	Age	Men	Women
18	21:44	24:16	40	22:14	24:59
19	21:28	24:04	41	22:24	25:11
20	21:15	24:01	42	22:34	25:24
21	21:06	24:00	43	22:45	25:38
22-28	21:01	24:00	44	22:55	25:54
29	21:02	24:00	45	23:06	26:16
30	21:04	24:01	46	23:17	26:29
31	21:07	24:02	47	23:28	26:49
32	21:11	24:05	48	23:39	27:09
33	21:16	24:08	49	23:50	27:29
34	21:21	24:12	50	24:02	27:50
35	21:28	24:17	51	24:13	28:12
36	21:36	24:24	52	24:25	28:34
37	21:45	24:31	53	24:37	28:57
38	21:55	24:39	54	24:50	29:21
39	22:04	24:49	55	25:02	29:45

USA Track & Field. Age-graded performance categories: http://www.usatf.org/statistics/calculators/agegrading/. 2014.

References

1. Matsuo M, Kaneko T. Chapter 1. The Chemistry of Reactive Oxygen Species and Related Free Radicals. In: Radak Z, ed. *Free Radicals in Exercise and Aging*. Champaign, IL: Human Kinetics; 2000:1-33.
2. Ji LL, Hollander J. Chapter 2. Antioxidant Defense: Effects of Aging and Exercise. In: Radak Z, ed. *Free Radicals in Exercise and Aging*. Champaign, IL: Human Kinetics; 2000:35-72.
3. Veskoukis AS, Nikolaidis MG, Kyparos A, Kouretas D. Blood reflects tissue oxidative stress depending on biomarker and tissue studied. *Free radical biology & medicine.* 2009;47(10):1371-1374.
4. Chen HJ, Chiu WL. Association between cigarette smoking and urinary excretion of 1,N2-ethenoguanine measured by isotope dilution liquid chromatography-electrospray ionization/tandem mass spectrometry. *Chemical research in toxicology.* 2005;18(10):1593-1599.
5. Margonis K, Fatouros IG, Jamurtas AZ, et al. Oxidative stress biomarkers responses to physical overtraining: implications for diagnosis. *Free radical biology & medicine.* 2007;43(6):901-910.
6. Chandwaney R, Leichtweis S, Leeuwenburgh C, Ji LL. Oxidative stress and mitochondrial function in skeletal muscle: Effects of aging and exercise training. *Age (Omaha).* 1998;21(3):109-117.
7. Atalay M, Lappalainen J, Sen CK. Dietary antioxidants for the athlete. *Current sports medicine reports.* 2006;5(4):182-186.

8. Arent SM, Pellegrino JK, Williams CA, Difabio DA, Greenwood JC. Nutritional supplementation, performance, and oxidative stress in college soccer players. *Journal of strength and conditioning research / National Strength & Conditioning Association.* 2010;24(4):1117-1124.

9. Nelson SK, Bose SK, Grunwald GK, Myhill P, McCord JM. The induction of human superoxide dismutase and catalase in vivo: a fundamentally new approach to antioxidant therapy. *Free radical biology & medicine.* 2006;40(2):341-347.

10. Burnham EL, McCord JM, Bose S, et al. Protandim does not influence alveolar epithelial permeability or intrapulmonary oxidative stress in human subjects with alcohol use disorders. *American journal of physiology. Lung cellular and molecular physiology.* 2012;302(7):L688-699.

11. Joddar B, Reen RK, Firstenberg MS, et al. Protandim attenuates intimal hyperplasia in human saphenous veins cultured ex vivo via a catalase-dependent pathway. *Free radical biology & medicine.* 2011;50(6):700-709.

12. Qureshi MM, McClure WC, Arevalo NL, et al. The Dietary Supplement Protandim Decreases Plasma Osteopontin and Improves Markers of Oxidative Stress in Muscular Dystrophy Mdx Mice. *Journal of dietary supplements.* 2010;7(2):159-178.

13. Robbins D, Gu X, Shi R, et al. The chemopreventive effects of Protandim: modulation of p53 mitochondrial translocation and apoptosis during skin carcinogenesis. *PLoS One.* 2010;5(7):e11902.

14. Liu J, Gu X, Robbins D, et al. Protandim, a fundamentally new antioxidant approach in chemoprevention using mouse two-stage skin carcinogenesis as a model. *PLoS One.* 2009;4(4):e5284.

15. Velmurugan K, Alam J, McCord JM, Pugazhenthi S. Synergistic induction of heme oxygenase-1 by the components of the antioxidant supplement Protandim. *Free radical biology & medicine.* 2009;46(3):430-440.

16. Scalzo R, Davis J, Beals J, et al. Oxidative stess is decreased with short-term Protandim use when piperine is substituted for ashwagandha [abstract]. *FASEB journal : official publication of the Federation of American Societies for Experimental Biology.* 2014;28:LB399.

17. Kyparos A, Vrabas IS, Nikolaidis MG, Riganas CS, Kouretas D. Increased oxidative stress blood markers in well-trained rowers following two thousand-meter rowing ergometer race. *Journal of strength and conditioning research / National Strength & Conditioning Association.* 2009;23(5):1418-1426.

18. Ware JE, Jr. SF-36 health survey update. *Spine.* 2000;25(24):3130-3139.

19. Ware JE, Jr., Gandek B. Overview of the SF-36 Health Survey and the International Quality of Life Assessment (IQOLA) Project. *Journal of clinical epidemiology.* 1998;51(11):903-912.

20. Ware JE, Kosinski M. Interpreting SF-36 summary health measures: a response. *Quality of life research : an international journal of quality of life aspects of treatment, care and rehabilitation.* 2001;10(5):405-413; discussion 415-420.

21. Skevington SM, Lotfy M, O'Connell KA, Group W. The World Health Organization's WHOQOL-BREF quality of life assessment: psychometric properties and results of the international field trial. A report from the WHOQOL group. *Quality of life research : an international journal of quality of life aspects of treatment, care and rehabilitation.* 2004;13(2):299-310.

22. Balady GJ, Chaitman B, Driscoll D, et al. Recommendations for cardiovascular screening, staffing, and emergency policies at health/fitness facilities. *Circulation.* 1998;97(22):2283-2293.

23. USA Track & Field. Age-graded performance categories: http://www.usatf.org/statistics/calculators/agegrading/. 2014.

24. Physical Activity Readiness Questinniare (PAR-Q): http://www.csep.ca/cmfiles/publications/parq/par-q.pdf. Candian Society for Exercise Physiology; 2002.

25. Jeukendrup AE, Currell K. Should time trial performance be predicted from three serial time-to-exhaustion tests? *Med Sci Sports Exerc.* 2005;37(10):1820; author reply 1821.

26. Mullins AL, van Rosendal SP, Briskey DR, Fassett RG, Wilson GR, Coombes JS. Variability in oxidative stress biomarkers following a maximal exercise test. *Biomarkers : biochemical indicators of exposure, response, and susceptibility to chemicals.* 2013;18(5):446-454.

27. Kyparos A, Riganas C, Nikolaidis MG, et al. The effect of exercise-induced hypoxemia on blood redox status in well-trained rowers. *Eur J Appl Physiol.* 2012;112(6):2073-2083.

28. Kabasakalis A, Kyparos A, Tsalis G, Loupos D, Pavlidou A, Kouretas D. Blood oxidative stress markers after ultramarathon swimming. *Journal of strength and conditioning research / National Strength & Conditioning Association.* 2011;25(3):805-811.
