# Supplementary material for: Correction: The Effect of Protandim® Supplementation on Athletic Performance and Oxidative Blood Markers in Runners
Source: PLoS One. 2020 Oct 23;15(10):e0241520. doi: 10.1371/journal.pone.0241520 (PMC7584197; doi:10.1371/journal.pone.0241520)
Supplement: S1 File — Pre and Post exercise blood values at baseline and at 88 days following supplementation. The baseline values were averaged over both baseline sessions. (DOCX) [file pone.0241520.s001.docx]

**Table 2. Pre and Post exercise blood values at baseline and at 88 days following supplementation.** The baseline values were averaged over both baseline sessions.

| **Damage** | **Reference** | **Pre-exercise** | **10 minutes Post-exercise** | **Change** |
| --- | --- | --- | --- | --- |
| **Lipid Peroxides (TBARs, μmol/L)** | ≤ 10 |  |  |  |
| *Protandim^®^ Group* |  |  |  |  |
| Baseline |  | 8.4 (2.1) | 8.5 (2.4) | +0.1 (1.7) |
| 88 days post-supplementation |  | 7.4 (2.2) | 7.5 (2.6) | +0.1 (1.2) |
| *Placebo Group* |  |  |  |  |
| Baseline |  | 7.9 (1.9) | 8.5 (2.5) | +0.6 (1.9) |
| 88 days post-supplementation |  | 7.7 (3.9) | 6.7 (2.5) | -1.0 (4.8) |
| **Protective Enzymes** | **Reference** | **Pre-exercise** | **10 minutes Post-exercise** | **Change** |
| **Superoxide dismutase* (SOD, U/g Hb x 1000)** | 5.3 to 16.7 |  |  |  |
| *Protandim^®^ Group* |  |  |  |  |
| Baseline |  | 11.5 (3.4) | 11.2 (3.5) | -0.3 (1.2) |
| 88 days post-supplementation |  | 20.2 (8.3) | 20.6 (7.4) | +0.4 (3.0) |
| *Placebo Group* |  |  |  |  |
| Baseline |  | 11.7 (3.7) | 12.0 (3.5) | +0.3 (1.1) |
| 88 days post-supplementation |  | 18.7 (6.2) | 19.2 (6.7) | +0.5 (3.4) |
|  | **Reference** | **Pre-exercise** | **10 minutes Post-exercise** | **Change** |
| **Glutathione Peroxidase (GPX, U/g Hb)*** | 20 to 38 |  |  |  |
| *Protandim^®^ Group* |  |  |  |  |
| Baseline |  | 27.7 (4.5) | 27.3 (4.3) | -0.4 (1.9) |
| 88 days post-supplementation |  | 30.7 (4.4) | 31.4 (5.2) | +0.7 (3.4) |
| *Placebo Group* |  |  |  |  |
| Baseline |  | 28.4 (7.4) | 28.6 (7.1) | +0.2 (1.3) |
| 88 days post-supplementation |  | 31.9 (7.8) | 31.4 (6.5) | -0.5 (2.8) |
| **Other** | **Reference** | **Pre-exercise** | **10 minutes Post-exercise** | **Change** |
| **Glucose levels (fasted) (mg/dL)*** | < 100 |  |  |  |
| *Protandim^®^ Group* |  |  |  |  |
| Baseline |  | 92 (5) | 166 (25) | +74 (27) |
| 88 days post-supplementation |  | 91 (5) | 158 (46) | +67 (44) |
| *Placebo Group* |  |  |  |  |
| Baseline |  | 90 (8) | 161 (35) | +71 (33) |
| 88 days post-supplementation |  | 91 (9) | 166 (43) | +75 (39) |
|  |  |  |  |  |
| **Reserve** | **Reference** | **Pre-exercise** | **10 minutes Post-exercise** | **Change** |
| **Glutathione (GSH) (μmol/L x 10)** | ≥ 66.9 |  |  |  |
| *Protandim^®^ Group* |  |  |  |  |
| Baseline |  | 103 (13) | 103 (11) | 0 (9) |
| 88 days post-supplementation |  | 104 (30) | 112 (18) | +8 (29) |
| *Placebo Group* |  |  |  |  |
| Baseline |  | 102 (19) | 100 (20) | -2 (8) |
| 88 days post-supplementation |  | 96 (18) | 96 (13) | 0 (16) |
|  | **Reference** | **Pre-exercise** | **10 minutes Post-exercise** | **Change** |
| **Total Antioxidant Capacity* (TAC, mmol/L)** | ≥ 0.54 |  |  |  |
| *Protandim^®^ Group* |  |  |  |  |
| Baseline |  | 0.86 (0.07) | 0.96 (0.07) | +0.11 (0.06) |
| 88 days post-supplementation |  | 0.80 (0.07) | 0.91 (0.09) | +0.11 (0.06) |
| *Placebo Group* |  |  |  |  |
| Baseline |  | 0.86 (0.06) | 0.97 (0.06) | +0.11 (0.04) |
| 88 days post-supplementation |  | 0.80 (0.06) | 0.90 (0.07) | +0.10 (0.03) |
|  | **Reference** | **Pre-exercise** | **10 minutes Post-exercise** | **Change** |
| **Cysteine (mg/dL)*** | 0.61 to 1.16 |  |  |  |
| *Protandim^®^ Group* |  |  |  |  |
| Baseline |  | 0.66 (0.14) | 0.67 (0.14) | +0.01 (0.13) |
| 88 days post-supplementation |  | 0.58 (0.13) | 0.67 (0.17) | +0.10 (0.14) |
| *Placebo Group* |  |  |  |  |
| Baseline |  | 0.63 (0.07) | 0.69 (0.08) | +0.06 (0.09) |
| 88 days post-supplementation |  | 0.61 (0.14) | 0.61 (0.14) | 0.00 (0.10) |
|  | **Reference** | **Pre-exercise** | **10 minutes Post-exercise** | **Change** |
| **Cystine (mg/dL)** | 1.6 to 3.2 |  |  |  |
| *Protandim^®^ Group* |  |  |  |  |
| Baseline |  | 2.1 (0.4) | 2.3 (0.4) | +0.2 (0.2) |
| 88 days post-supplementation |  | 2.1 (0.4) | 2.1 (0.5) | 0.0 (0.5) |
| *Placebo Group* |  |  |  |  |
| Baseline |  | 2.2 (0.3) | 2.3 (0.2) | 0.0 (0.3) |
| 88 days post-supplementation |  | 2.1 (0.3) | 2.3 (0.4) | +0.2 (0.2) |
|  | **Reference** | **Pre-exercise** | **10 minutes Post-exercise** | **Change** |
| **Cysteine to Cystine ratio#** | 0.23 to 0.53 |  |  |  |
| *Protandim^®^ Group* |  |  |  |  |
| Baseline |  | 0.33 (0.12) | 0.31 (0.13) | -0.01 (0.08) |
| 88 days post-supplementation |  | 0.29 (0.12) | 0.34 (0.15) | +0.05(0.08) |
| *Placebo Group* |  |  |  |  |
| Baseline |  | 0.29 (0.07) | 0.31 (0.06) | +0.02 (0.08) |
| 88 days post-supplementation |  | 0.29 (0.07) | 0.27 (0.07) | -0.02(0.06) |
|  | **Reference** | **Pre-exercise** | **10 minutes Post-exercise** | **Change** |
| **Sulfate (mg/dL)** | 3.0 to 5.9 |  |  |  |
| *Protandim^®^ Group* |  |  |  |  |
| Baseline |  | 3.8 (0.5) | 4.0 (0.6) | +0.2 (0.6) |
| 88 days post-supplementation |  | 3.9 (0.6) | 4.1 (0.8) | +0.2 (0.4) |
| *Placebo Group* |  |  |  |  |
| Baseline |  | 3.9 (0.9) | 4.4 (1.1) | +0.5 (1.0 ) |
| 88 days post-supplementation |  | 3.8 (0.7) | 4.1 (0.8) | +0.3 (0.6) |
|  | **Reference** | **Pre-exercise** | **10 minutes Post-exercise** | **Change** |
| **Cysteine to Sulfate ratio** | 0.12 to 0.32 |  |  |  |
| *Protandim^®^ Group* |  |  |  |  |
| Baseline |  | 0.18 (0.05) | 0.18 (0.05) | 0.00 (0.04) |
| 88 days post-supplementation |  | 0.15 (0.04) | 0.17 (0.04) | +0.02 (0.04) |
| *Placebo Group* |  |  |  |  |
| Baseline |  | 0.17 (0.14) | 0.17 (0.05) | 0.00 (0.04) |
| 88 days post-supplementation |  | 0.16 (0.05) | 0.15 (0.04) | -0.01 (0.03) |
| Mean (SD). There were 16 and 19 subjects in the Protandim Group and Placebo group, respectively, that completed all sessions. This was assessed by a 2 x 4 repeated measures ANOVA (2 groups, 4 time-points: pre and post exercise at baseline, and pre and post exercise post-supplementation). An asterisk* shows that a main effect of Time was present, even after adjusting for multiple comparisons. A number symbol# shows that a Group x Time interaction effect was present (p < 0.05). The reference range was provided to us by Genova Diagnostics. | | | | |
