# Supplementary figures and images for: Correction: The Effect of Protandim® Supplementation on Athletic Performance and Oxidative Blood Markers in Runners
Source: PLoS One. 2020 Oct 23;15(10):e0241520. doi: 10.1371/journal.pone.0241520 (PMC7584197; doi:10.1371/journal.pone.0241520)

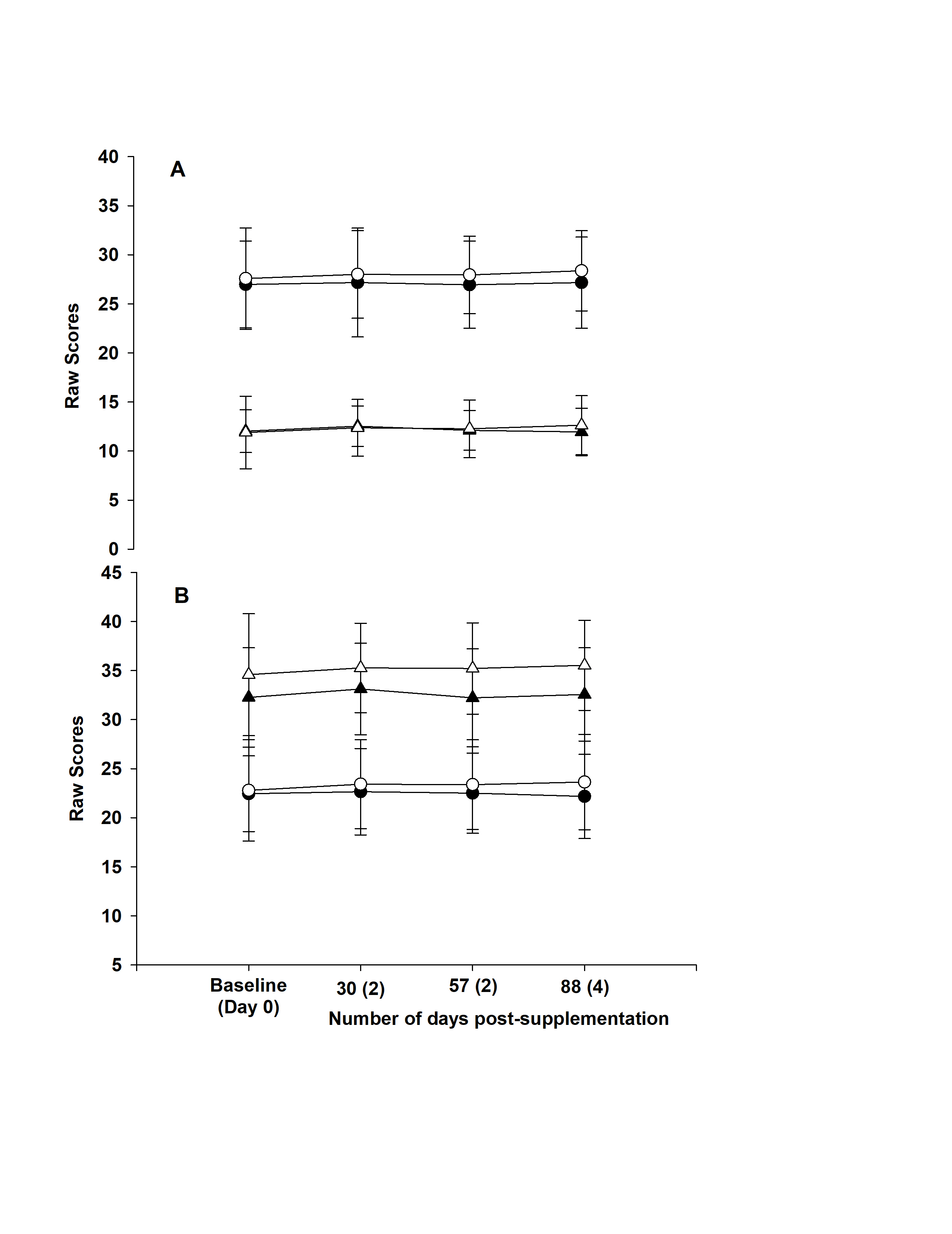

Supplement: S3 File — These are the World Health Organization Quality of life (BREF) raw scores. Upper panel A, circles, represent Physical Health scores, triangles represent Social Relationship scores. Lower panel B, circles, represent Psychological Health scores, triangles represent Environment scores. (Black = Protandim®, White = Placebo). Mean values represented by circles and triangles, error bars represent SD. There was no statistical difference between groups or between timepoints after Bonferroni-adjusting for multiple comparisons. (TIF) [file pone.0241520.s003.tif]
